# Successive paired electrochemical late-stage modification of niclosamide a common anthelmintic drug. A green protocol for the synthesis of new drug-like molecules†

**DOI:** 10.1039/d5ra02025e

**Published:** 2025-05-29

**Authors:** Haniya Rezaei, Davood Nematollahi, Niloofar Mohamadighader, Farideh Lotfipour

**Affiliations:** a Faculty of Chemistry and Petroleum Sciences, Bu-Ali Sina University Hamedan Iran 65178-38683 nemat@basu.ac.ir; b Planet Chemistry Research Center, Bu-Ali Sina University Hamedan Iran

## Abstract

Drugs based on salicylanilides such as niclosamide are of particular interest to medicinal chemistry researchers. They exhibit a wide range of biological activities, including anticancer and antiviral activities. Niclosamide is a common oral anthelmintic that has the potential to be an antiviral and anticancer drug. However, two characteristics of it, including poor oral bioavailability and high cytotoxicity, have limited its use. The synthesis of new niclosamide analogs is an attempt to overcome these limitations. The electrochemical behavior of niclosamide shows that the drug can be reduced and then oxidized at the cathode and anode, respectively. This property, along with the special capabilities of electrosynthesis methods, makes it possible to obtain unique niclosamide analogs. In this study, novel niclosamide analogs were synthesized *via* successive paired electrolysis of niclosamide in the presence of arylsulfinic acids as nucleophiles. The results show that niclosamide is converted to the desired product (5-chloro-*N*-(2-chloro-4-(phenylsulfonamido)phenyl)-2-hydroxy benzamide) after reduction and oxidation steps and reaction with the nucleophile. In the synthesized niclosamide analogs, a sulfonamide moiety is attached to the drug molecule. This work presents a green method for the synthesis of new niclosamide analogs without the need for catalysts, reductants or oxidants under mild conditions in a one-pot process.

## Abstract 2

Novel niclosamide analogs (LSP) were synthesized *via* successive paired electrolysis of niclosamide (NIC) in the presence of arylsulfinic acids. This work presents a green method under mild conditions in a one-pot process.

## Graphical abstract

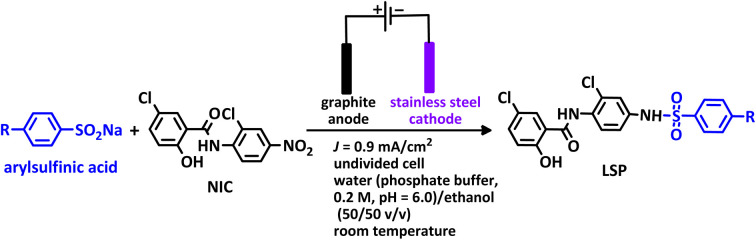

## Introduction

The process of discovery and clinical use of a new drug that has a new chemical structure is very long and may take 12 to 15 years. To solve this problem, one strategy is late-stage modification of drugs that are currently on the market to treat other diseases. Niclosamide (NIC) is one of these drugs that has been studied in this way due to its special characteristics. This drug is an anthelmintic drug effective against various intestinal tapeworms. It is particularly suitable for adult intestinal tapeworm infections, as it can be administered orally in a single daily dose or in multiple doses throughout the day. Since anthelmintic drugs are usually designed to have low absorption when taken orally, the very limited bioavailability of NIC hinders its direct use as an antiviral and anticancer drug.^1^ However, recent studies have shown that NIC analogs have a wide range of clinical applications as a potential anticancer and antiviral agent.^2–7^ In this regard, in 2024, Ma *et al.* synthesized some niclosamide derivatives by replacing the nitro group with sulfanyl groups.^8^ They reported that some of the synthesized derivatives exhibited distinct anticancer and antifungal activities. Berry *et al.* in 2024, synthesized a series of niclosamide analogs by replacing various groups with nitro group to perform structure activity relationship (SAR) and investigated their synergistic effect with colistin against Gram-negative bacteria.^9^ They reported that some of the synthesized derivatives reduced toxicity while maintaining the ability of niclosamide to synergize with colistin against Gram-negative bacteria. Li *et al.* designed and synthesized some NIC analogs in 2023 and showed that a large number of synthesized derivatives had anti-SARS-CoV-2 activity, lower cytotoxicity, and better pharmacokinetics.^10^ In the same year, Kang *et al.* synthesized a new series of NIC analogs and examined their activity against enzalutamide-resistant prostate cancer cells. They found that this property was equivalent or improved in a number of niclosamide analogues.^11^ Also in 2023, Lal *et al.* synthesized halogen-rich salicylanilides including niclosamide and used them as antileishmanial agents.^12^ They reported that one of the synthesized derivatives could be a promising lead candidate with the potential to become a *Leishmania* drug. In 2021, Shamim *et al.* conducted structure activity relationship (SAR) studies on NIC for inhibition of Zika virus and SARS-CoV-2 infection.^13^ They found that replacing bromine with chlorine in the phenolic ring maintained the drug's potency while providing better drug-like properties. They also reported that –CF_3_ or –CN groups could replace the nitro group. These papers, along with a number of others not discussed here, demonstrate the importance of synthesizing new NIC derivatives.

**Fig. 1:**
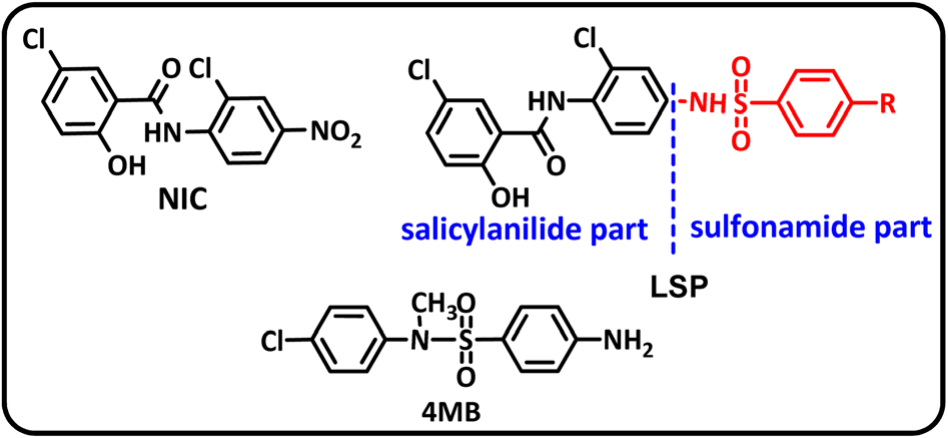
The structures of niclosamide (NIC) and late-stage products (LSP).

In addition to the above, here we would like to review the electrochemical studies conducted on NIC. In 2024, Darvishi *et al.* studied the cyclic voltammogram of NIC and concluded that NIC is initially reduced to its hydroxylamine derivative and as a result the anodic and cathodic peaks observed in the anodic scan are related to the oxidation of the hydroxylamine derivative to nitroso derivative and *vice versa*.^14^ In 2023, Liu *et al.* also recorded cyclic voltammograms of NIC and reported similar reaction mechanism.^15^ Li *et al.* in 2022, also investigated the electrochemical behavior of NIC and reported a mechanism similar to the previous two cases.^16^ This mechanism has been repeated in exactly the same way in many similar papers over the years in various journals.^17–23^ In this paper, we take a different look at the reduction mechanism of NIC and other peaks in its cyclic voltammogram. Therefore, another aim of this research is to report a more probable mechanism for the electrochemical behavior of NIC as well as to complement other electrochemical data available in the literature. With regard to the above objectives, first, the electrochemical behavior of NIC alone and also in the presence of arylsulfinic acids as unique nucleophiles was investigated, and in addition to providing complete information about the electrochemical behavior of NIC, the necessary conditions for the late-stage modification of NIC were also identified. In the next step, using the obtained electrochemical data, NIC was electrolyzed in the presence of arylsulfinic acids and the synthesized products (LSP) were identified after purification (Fig. 1). The late-stage modification of NIC has led to the synthesis of unique molecules (LSP) that have two components. The main building block, salicylanilide, is the main structural skeleton of NIC and has proven medicinal properties.^24^ The second component is a benzene sulfonamide component that has known medicinal properties.^25^ This part, together with the benzene ring attached to it, forms the benzenesulfonanilide scaffold, whose derivatives, such as 4-amino-4′-chloro-*N*-methylbenzene sulfonanilide (4MB), have medicinal properties.^26^

Combining these two moieties into one molecule provides a completely new compound with many unique properties, raising the expectation that the synthesized products will have specific pharmacological and biological properties. Therefore, we decided to synthesize molecules (LSP) containing both of the aforementioned pharmacologically active moieties in a single framework. The synthesis of these molecules (LSP) was carried out in a one-pot process in simple electrochemical cells under green conditions using the galvanostatic technique, in a water/ethanol mixture, in the absence of catalysts and chemical oxidants, with good yield. It is important to mention at the end of this section that no electrochemical method has been reported to date for the late-stage modification of NIC.

## Results and discussion

### Electrochemical study of niclosamaide

**Fig. 2:**
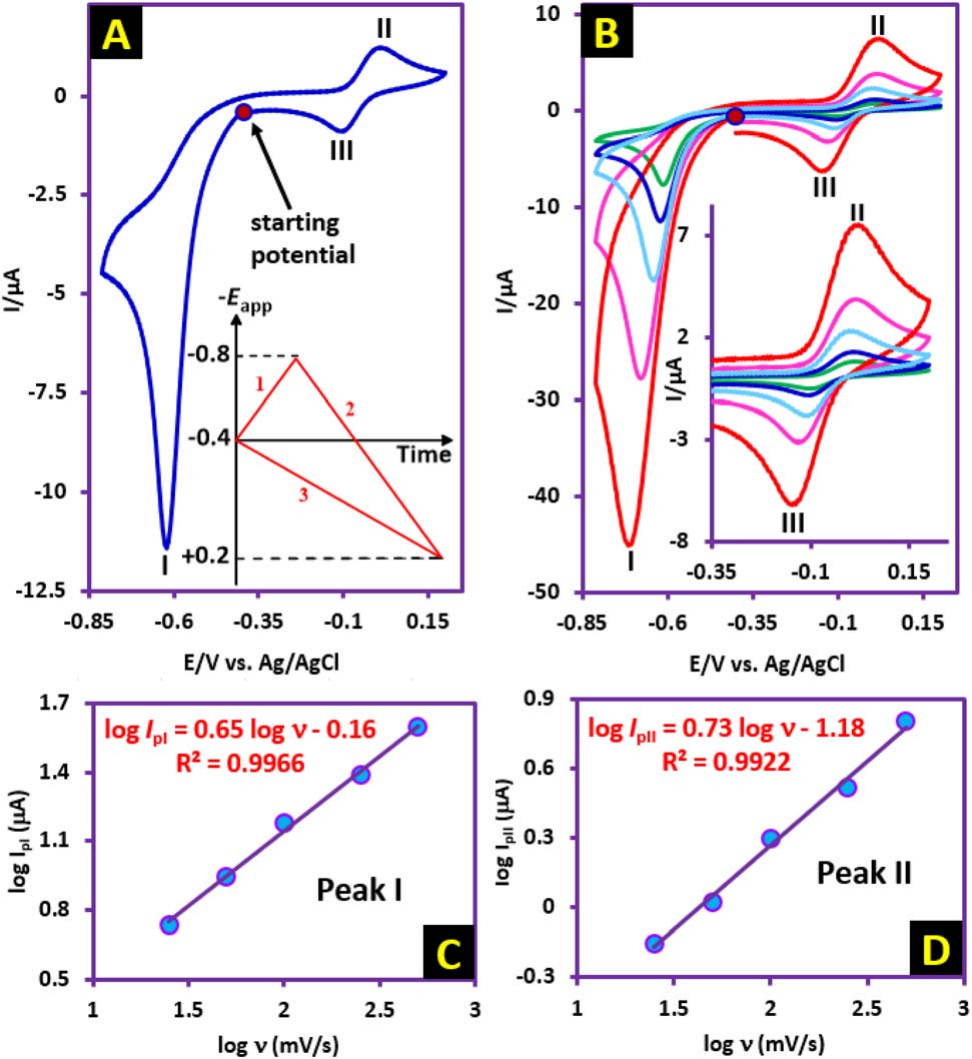
Part (A) Cyclic voltammogram of 1.0 mM NIC at scan rate of 50 mV s^−1^. Part (B) Cyclic voltammograms of NIC (1.0 mM) at different scan rates. Scan rates are: 25, 50, 100, 250 and 500 mV s^−1^. Working electrode : glassy carbon electrode. Solvent : water (phosphate buffer, pH, 6.0, *c* = 0.2 M)/ethanol (50/50 v/v) mixture at GC electrode, at room temperature. Part (A) inset: Potential scanning program. Part (B) inset: Expanded anodic part. Part (C) log *I*^I^_p_*vs.* log *ν* plot for cathodic peak I. Part (D) log *I*^II^_p_*vs.* log *ν* plot for anodic peak II.

**Scheme 1:**
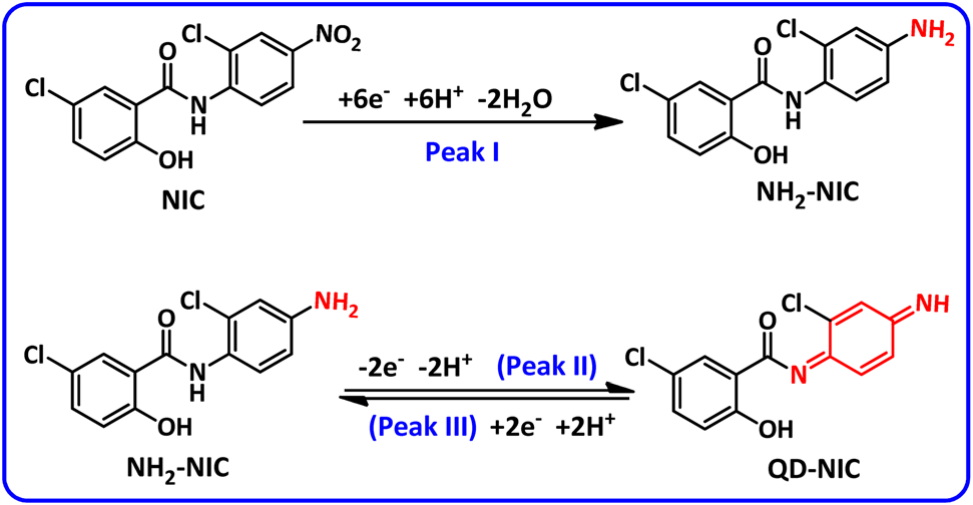
Proposed mechanism for the redox behavior of NIC in acidic and neutral solutions.

In this section, to gain further insight into the reduction mechanism of NIC, we have investigated the electrochemical behavior of NIC using cyclic voltammetry (CV). Fig. 2, part (A), inset, shows the three steps of potential scanning. Accordingly, the potential is first scanned in the cathodic direction from −0.4 V to −0.8 V (*vs.* 3 M KCl Ag/AgCl), and then the scanning direction is reversed to the anodic direction to +0.2 V, and in the final stage, it is scanned again towards the cathodic direction to −0.4 V. In the first cathodic scan, a well-defined cathodic peak (I) appears at −0.62 V, which is attributed to the six-electron reduction of NIC nitro group to the corresponding amino group (formation of NH_2_–NIC) (Fig. 2, part (A)).^27^ By changing the potential direction to the anodic direction (second stage of scanning), an anodic peak (II) appears at 0.01 V. This peak corresponds to the oxidation of NH_2_–NIC to the corresponding quinonediimine (QD–NIC). In the third scan stage (cathodic scan), a new cathodic peak (III) appears at potential −0.10 V, which corresponds to the two-electron reduction of QD–NIC to NH_2_–NIC (Fig. 2, part (A)). The electrode processes associated with peaks I, II, and III are shown in Scheme 1. Fig. 2, part (B) and its inset show the effect of potential scan rate on the voltammogram of NIC. Several points can be concluded from this figure. First, the peak current ratio (*I*^II^_p_/*I*^III^_p_) decreases from 1.5 at a scan rate of 25 mV s^−1^ to 1.3 at a scan rate of 500 mV s^−1^, indicating that the quinonediimine (QD–NIC) produced under our experimental conditions and on the time scale of cyclic voltammetry is relatively unstable. It seems that QD–NIC, like other quinonediimines, participates in processes such as hydroxylation or dimerization.^28–35^

The second point is the increase in Δ*E*_p_(*E*^II^_p_ − *E*^III^_p_) from 0.11 V at a scan rate of 25 mV s^−1^ to 0.15 V at a scan rate of 500 mV s^−1^. This result confirms the quasi-reversible nature of the NH_2_–NIC/QD–NIC redox process and the enhancement of this property at higher scan rates. The third point concerns the adsorptive or diffusive nature of the redox processes observed in Fig. 2. The plot of log *I*^I^_p_*vs.* log *ν* for cathodic peak I (reduction of nitro group) is shown in Fig. 2, part (C). The slope of the line in these diagrams indicates whether the redox process is diffusion-controlled (when slope is 0.5) or adsorption-controlled (when slope is 1).^36^ Looking at the slope of the line (0.65), it is clear that the process governing the reduction of the nitro group (under our experimental conditions and on the surface of the glassy carbon electrode), is a diffusion–adsorption process. The presence of abundant functional groups like –OH, –NO_2_, –Cl, –CO and –NH in the structure of NIC and the interactions such as hydrogen bonding that they have with the solvent is major factor in the relatively diffusive nature of the reduction process of this molecule. In addition, the plot of log *I*^II^_p_*vs.* log *ν* for anodic peak II (oxidation of NH_2_–NIC) is shown in Fig. 2, part (D). In this case, the slope of the line is 0.73. Comparing these two slopes shows that the adsorption activity of NH_2_–NIC is slightly higher than that of NIC.

### The effects of pH

**Fig. 3:**
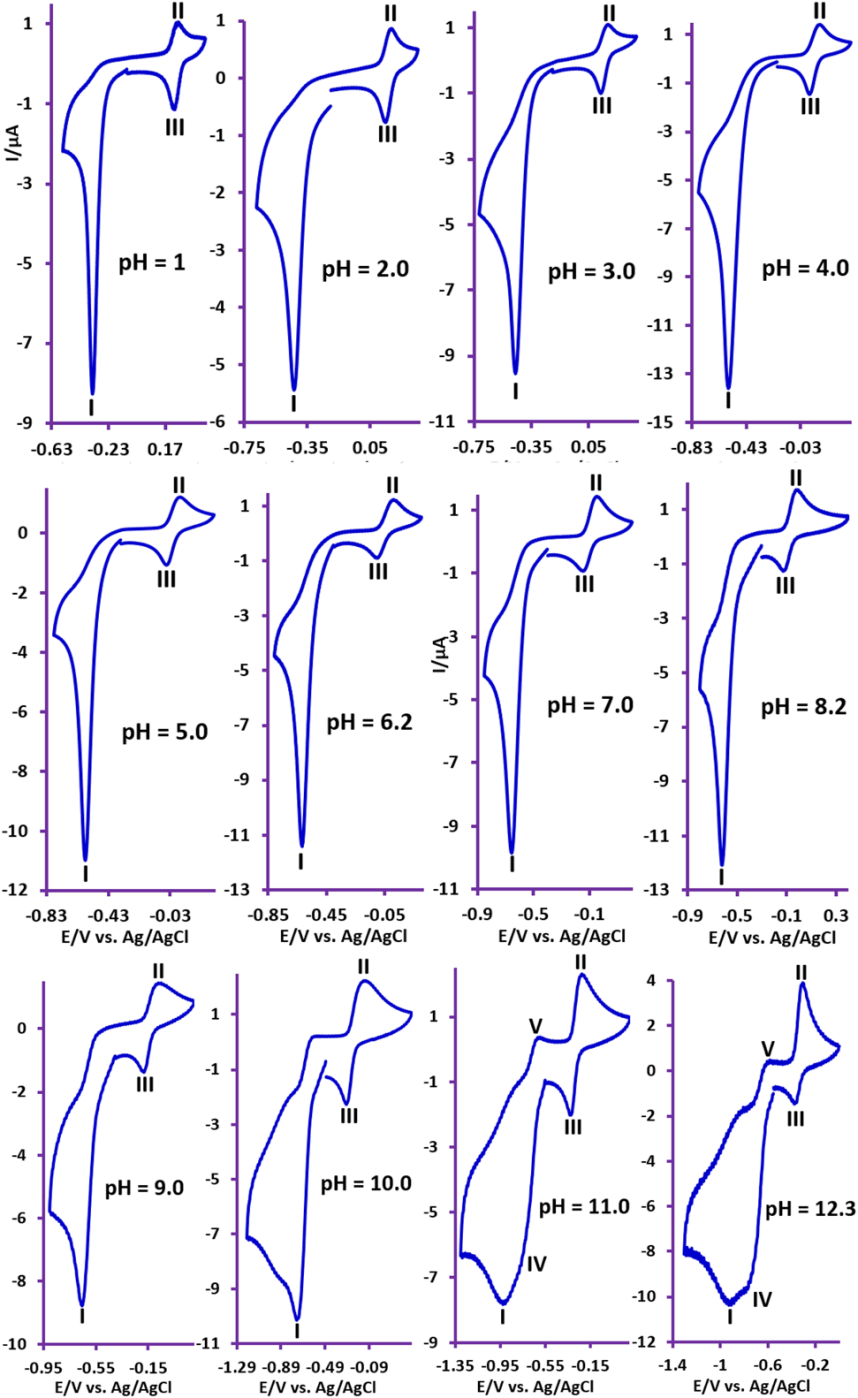
Cyclic voltammograms of NIC (1 mM) at glassy carbon electrode, at scan rate of 50 mV s^−1^, in water (with different pH values)/ethanol (50/50, v/v) mixture at room temperature. For pH = 1, perchloric acid 0.1 M; for pH values of 2.0, 3.0, 6.2, 7.0, 8.2 and 12.3, phosphate buffer; for pH values of 4.0 and 5.0, acetate buffer and for pH values of 9.0, 10.0 and 11.0, carbonate buffer were used.

Given the significant effect of environmental pH on the redox reactions of organic compounds, in this section we will examine the effect of solution pH in a wide range of pH (1–12) on the redox processes of NIC (Fig. 3).

**Scheme 2:**
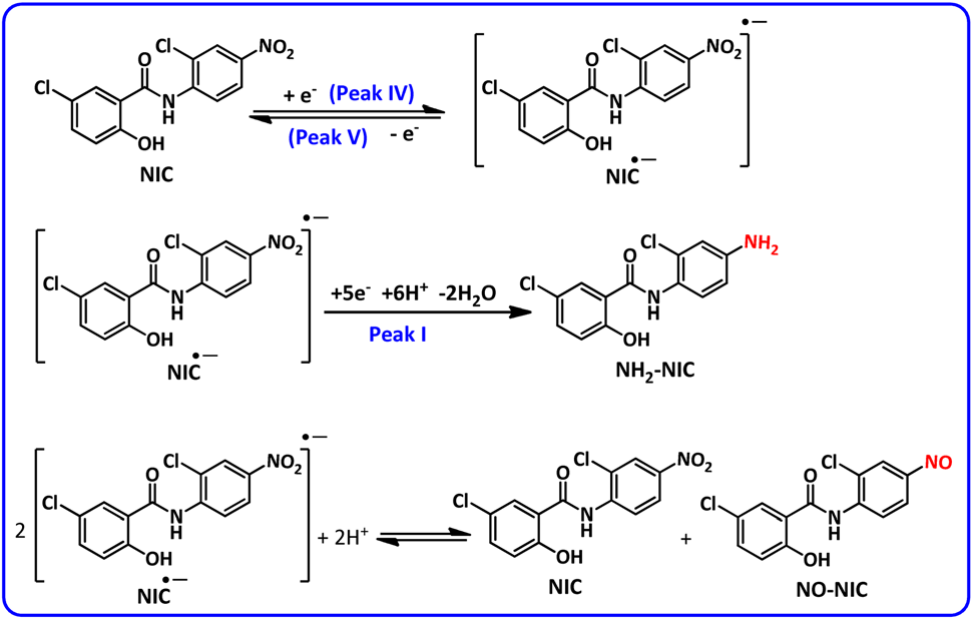
Proposed mechanism for the reduction of NIC in alkaline solutions.

The results show that with increasing pH, all peaks shift to less positive potentials. At acidic, neutral and slightly alkaline pHs, the shape of the voltammograms does not change and the voltammograms contain peaks I, II and III, while with increasing solution alkalinity, a new cathodic/anodic couple peak (IV and V) appears at less negative potentials than *E*^I^_p_. According to a previous report,^37^ peak IV corresponds to the one-electron reduction of NIC to its radical anion 
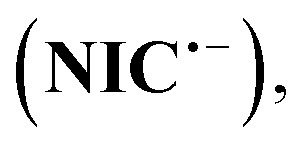
 and peak V is the counterpart of peak IV and corresponds to the oxidation of the radical anion 
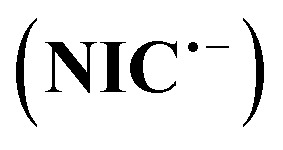
 to NIC (Scheme 2). 
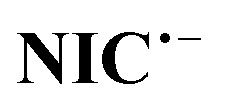
 is an unstable intermediate and, by participating in the disproportionation reaction in acidic environments, is rapidly converted to NIC and nitroso derivative of niclosamide (NO–NIC) (Scheme 2), and therefore the peak corresponding to its reduction (peak IV) is not observed. In contrast, in alkaline solutions, the disproportionation reaction rate is slow and therefore the peak corresponding to the reduction of NIC to 
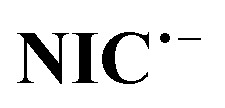
 is observed. In such environments, the nitro reduction process is divided into two processes: one-electron and five-electron (Scheme 2).

**Fig. 4:**
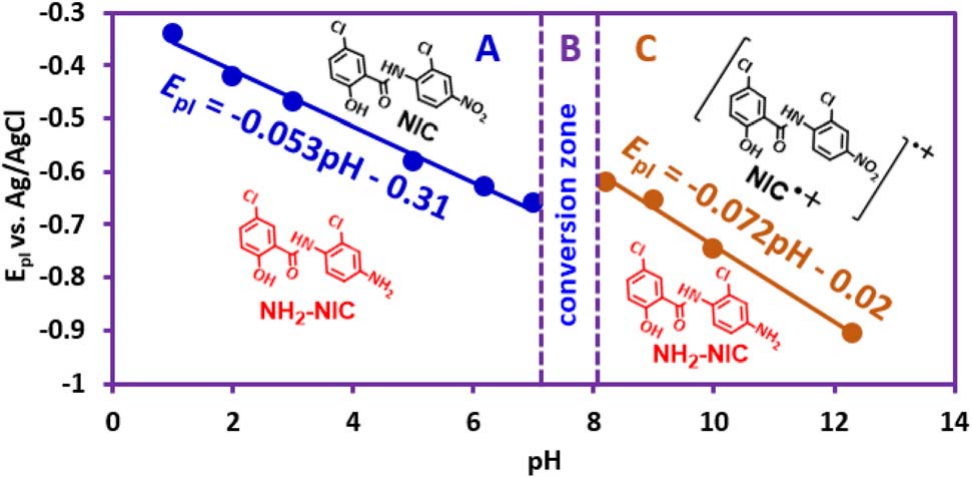
The *E*-pH diagram for reduction of nitro group.

Fig. 4 shows that *E*^I^_p_ is pH dependent and shifts to more negative potentials with increasing pH. The diagram consists of two distinct lines with different slopes. In other words, Fig. 4 consists of three regions, A, B, and C. The slope of the line in region A is 53 mV pH^−1^, which is close to a theoretical value of 59 mV pH^−1^ for a process with equal numbers of electrons and protons. The process of this line is shown in Scheme 1. In this pH region (pH < 7), NIC is reduced to NH_2_–NIC by gaining six electrons and six protons. The slope of the line in region C is 72 mV pH^−1^, which is equal to the theoretical slope for a five-electron/six-proton process. The process of this line is shown in Scheme 2. In this pH region (pH > 8), 
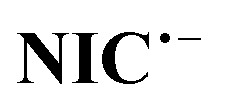
 is reduced to NH_2_–NIC by gaining five electrons and six protons. Region B is an intermediate region between regions A and C. This region is actually a conversion zone. In this pH range, the rate of the disproportionation reaction decreases to such an extent that the six-electron process of amine group reduction is converted into two one- and five-electron processes.

### Electrochemical study of NIC in the presence of arylsulfinic acids

**Fig. 5:**
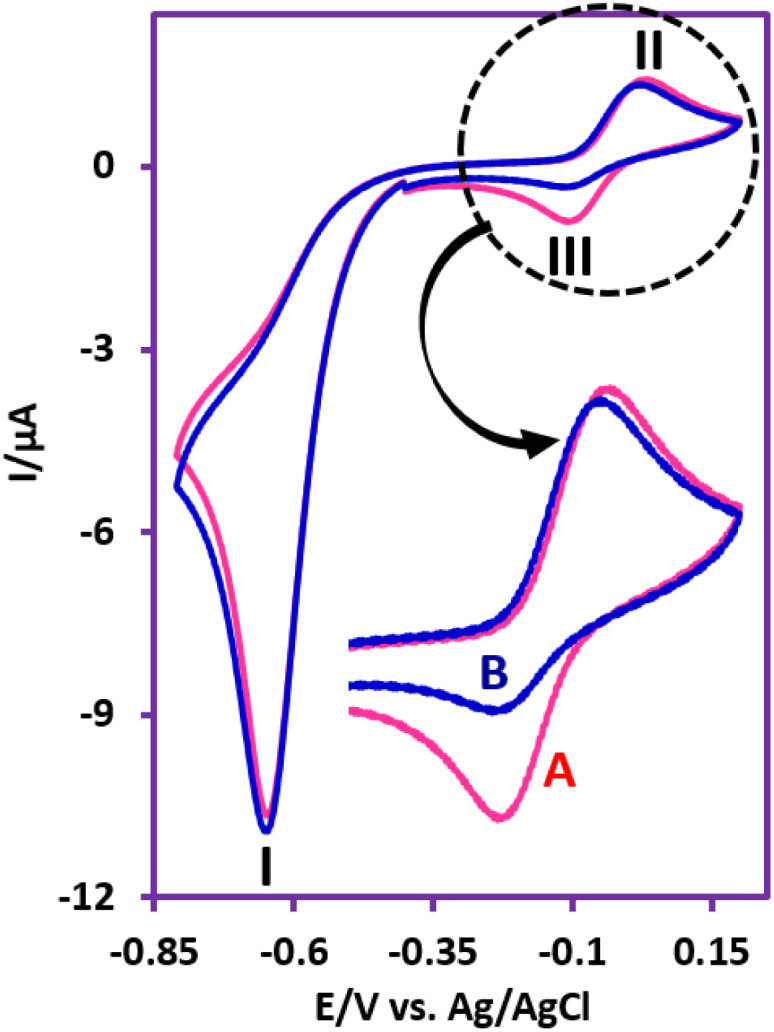
(A) Cyclic voltammogram of NIC (1.0 mM) at GC electrode, in water (phosphate buffer, *c* = 0.2 M, pH = 6.0)/ethanol (50/50 v/v). (B) as (A) in the presence of *p*-toluenesulfinic acid sodium salt (2.0 mM). Scan rate of 50 mV s^−1^.

**Scheme 3:**
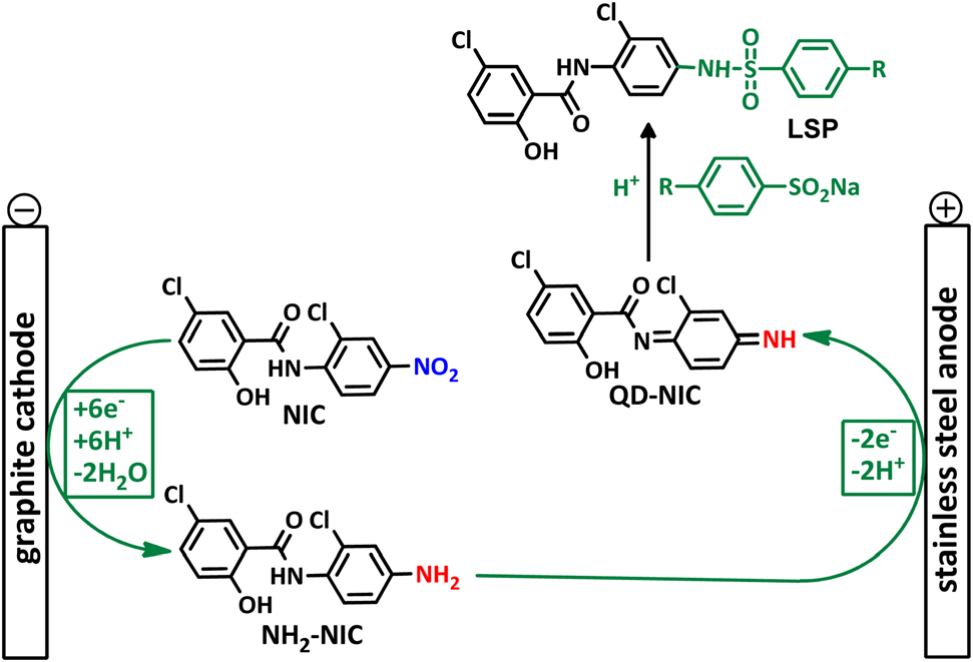
Successive paired electrochemical late-stage modification of NIC.

To study the electrochemical behavior of NIC in the presence of *p*-toluenesulfinic acid, the cyclic voltammogram of a mixture of NIC and *p*-toluenesulfinic acid was recorded and compared with the cyclic voltammogram of NIC in the absence of *p*-toluenesulfinic acid (Fig. 5). The most important change in the presence of *p*-toluenesulfinic acid is the decrease in the current corresponding to cathodic peak III (*I*^III^_p_). This finding suggests that QD–NIC produced from the oxidation of NH_2_–NIC reacts with *p*-toluenesulfinic acid under the present experimental conditions. Based on the available electrochemical data as well as spectroscopic data obtained from the analysis of products obtained from the electrolysis of NIC in the presence of arylsulfinic acids, the following mechanism has been proposed for the electrolysis of NIC in the presence of arylsulfinic acids (Scheme 3).

According to the proposed mechanism, initially, NIC is converted to NH_2_–NIC*via* a six-electron reduction at the cathode. Then, at the anode, the NH_2_–NIC loses two electrons and two protons and is oxidized to QD–NIC. The formed quinonediimine (QD–NIC) acts as a Michael acceptor and reacts with arylsulfinic acids to form the final product (LSP). We have introduced this type of pair electrosynthesis, which is different from the four previously known types (parallel paired, convergent paired, divergent paired and linear paired electrosynthesis), as successive paired electrosynthesis.^35^ This method saves 25% energy consumption in reactions such as the late-stage modification of niclosamide compared to conventional electrochemical methods. The use of both cathodic and anodic processes and the lack of need for reducing and oxidizing chemical agents to produce the product are unique advantages of the proposed method.

This method is superior in terms of energy consumption compared to conventional electrosynthesis methods, as it will lead to a reduction of about 25% in energy consumption in the synthesis of LSP derivatives. It should be noted, that if the number of electrons passing through the cathode and anode are equal, the reduction in energy consumption can even reach 50%. As shown in Scheme 3, the late-stage functionalization of NIC has led to the synthesis of LSP derivatives that possess two pharmacologically active moieties. The first moiety is salicylanilide, which is the main structural skeleton of NIC and has proven pharmacological properties.^16^ The second moiety is a benzene sulfonamide part, whose medicinal properties have also been proven.^17,18^ The presence of these two moieties in a single framework can improve the pharmaceutical properties of the produced molecules due to their synergy with each other.

### Optimization of effective parameters

In this section, in order to evaluate the performance of the proposed method for the synthesis of NIC derivatives (LSP1–3), the parameters affecting the yield and purity of the products, including current density, anode and cathode materials, organic solvent, solution pH were optimized (Table 1). In this work, the “*one-factor-at-a-time*” method was used to optimize the parameters, in which one factor is changed at a time while other factors are kept constant. In constant current electrosynthesis, one of the most important factors affecting the yield and purity of products is the applied current density. Entries 1 to 5 show the effect of the applied current density on the yield of LSP2. These experiments were conducted by keeping other effective factors constant and only the current density was changed. As can be seen, as the current density increased from 0.22 mA cm^−2^ to 0.90 mA cm^−2^, the yield of LSP2 increased from 5 to 63%, but further increases in current density caused a decrease in yield. Therefore, the optimal current density for LSP2 synthesis is 0.90 mA cm^−2^. As the current density increases, the potential (or rather, the overvoltage) applied to the electrodes increases and the desired cathodic and anodic reactions occur at a more appropriate rate, resulting in increased product production yield. However, further increase in current density causes an increase in side reactions such as reduction and/or oxidation of the solvent, over-reduction and/or oxidation of the product, and consequently reduce product yield.

**Table 1:** Optimization of effective parameters in the synthesis of LSP2^a^

Entry	Current density (mA cm^−2^)	Anode	Cathode	Water pH	Electricity (C)	Isolated yield [%]
1	0.22	Stainless steel	Graphite	6.0	285	5
2	0.45	Stainless steel	Graphite	6.0	285	32
**3**	**0.90**	**Stainless steel**	**Graphite**	**6.0**	**285**	**63**
4	1.36	Stainless steel	Graphite	6.0	285	42
5	1.80	Stainless steel	Graphite	6.0	285	27
6	0.90	Graphite	Graphite	6.0	285	48
7	0.90	Stainless steel	Stainless steel	6.0	285	15
8	0.90	Cu	Graphite	6.0	285	13
9	0.90	Graphite	Fe	6.0	285	61
10	0.90	Graphite	Cu	6.0	285	53
11	0.90	Stainless steel	Graphite	2.0	285	11
12	0.90	Stainless steel	Graphite	4.0	285	22
13	0.90	Stainless steel	Graphite	8.0	285	39

a In an undivided cylindrical glass cell (100 mL) containing NIC (0.5 mmol) and arylsulfinic acid (1 mmol) in water/ethanol (50/50 v/v) mixture.

The cathode and anode materials are effective in the generation of NH_2_–NIC and QD–NIC, respectively, and therefore require optimization. In entries 3 and 6 to 10, the effect of electrode materials on product yield is investigated. The results show that the highest yield is achieved when stainless steel is used for the anode and graphite for the cathode (entry 3). One of the main influential parameters that significantly affect the yield and purity of LSP2 is the pH of the solution. NIC reduction and NH_2_–NIC oxidation are pH-dependent processes. On the other hand, nucleophile deprotonation or electrophile protonation is also affected by the solution pH, and all these processes affect the yield and purity of the product. The effect of pH on product yield is shown in entries 3 and 11 to 13.

This study shows that the highest yield is achieved at pH 6. The protonation of NH_2_–NIC and the relative impairment of its oxidation, as well as the relative impairment of *p*-toluenesulfinic acid deprotonation, are factors that reduce the yield in more acidic solutions than pH 6. On the other hand, relative impairment in NIC reduction and the occurrence of side reactions such as hydroxylation or dimerization are among the processes that can reduce product yield in alkaline solutions.

## Conclusions

In this work, a new series of NIC analogs have been rationally designed and synthesized in the hope of their more promising activity than the parent drug. This method becomes more valuable when carried out electrochemically, and especially when the electrochemical method used is a paired and environmentally friendly method. This study presents a very simple method for the synthesis of new NIC derivatives using a simple cell equipped with conventional graphite and stainless-steel electrodes without the need for catalysts or oxidants at ambient temperature and pressure, in a water/ethanol mixture, under mild conditions. The products have two pharmacologically active moieties. The first moiety is salicylanilide, which is the main structural skeleton of NIC and the second moiety is a benzene sulfonamide part. The pharmacological properties of both moieties have already been proven, and it seems that the presence of these two moieties in a single framework can improve the pharmacological properties of the produced molecules (LSP) due to their synergy with each other. In this work, we used benzenesulfinic acid and two other derivatives *p*-toluenesulfinic acid (with an electron-donating substituent) and *p*-bromobenzenesulfinic acid (with an electron-withdrawing substituent) as nucleophiles and were able to synthesize the desired products (LSP) with a yield of 58 to 63%. In addition to what was mentioned above, in this work, we presented a more complete and plausible mechanism for the electrochemical behavior of NIC and also complemented other electrochemical data available in the literature. These data are valuable for more advanced studies in the future and can be used in the design and synthesis of new analogs of NIC.

## Experimental section

### Apparatus and reagents

NIC was extracted in ethanol from tablets commercially available (from Iran's Zagros Farmed Pharmaceutical Company). The chemicals for the preparation of buffer solutions including phosphoric acid, acetic acid, hydrochloric acid, sodium carbonate, sodium hydrogen carbonate and sodium hydroxide were obtained from Merck and were used without purification. The solvents used in this study including, ethyl acetate, *n*-hexane, ethanol was obtained from Merck and Sigma-Aldrich companies and were used without purification. 4-Bromobenzenesulfinic acid was synthesized in our laboratory in two steps from 4-bromobenzenethiol according to the methods described by Bahrami^38^ and Liu.^39^ Other sulfinic acid derivatives (benzene and toluene) and 4-bromobenzenethiol were obtained from Merck. Autolab PGSTAT302N potentiostat/galvanostat was used to perform cyclic voltammetry measurements of NIC. A three-electrode system consisting of a glassy carbon disc working electrode (2.8 mm diameter), an Ag/AgCl reference electrode (3 M KCl), and a stainless-steel wire counter electrode was used for voltammetric measurements. The glassy carbon electrode was polished with alumina slurry. All electrodes are made by Azar Electrode Company (Iran). A Dazheng ps-303D power supply was used for electrosynthesis at constant current condition. The electrochemical synthesis was carried out in a simple cylindrical glass cell (100 mL), containing water/ethanol (50/50 v/v) mixture, equipped with a set of four graphite rods (each one 5.5 cm long and 10 mm in diameter) as anodes and a stainless-steel rod as cathode. To distribute the potential uniformly across the electrodes, the stainless-steel electrode is placed in the center and the graphite electrodes are placed at equal distances around it. The low solubility of NIC in water prompted us to use ethanol as a co-solvent to increase the solubility of NIC.

The FTIR spectra of the products were recorded from 4000 to 400 cm^−1^ using a KBr disk on an FTIR spectrometer (PerkinElmer GX FT-IR). A Bruker Avance DRX 300 spectrometer was used for NMR experiments. The spectrometer operates at 300 MHz for protons and 75 MHz for carbon. All spectra were recorded in DMSO-*d*_6_ and chemical shifts are reported in ppm (*δ*) at 298 K. The mass spectra recorded by MS model: 5975c VL MSD with Tripe-Axis Detector. The electron ionization was performed at 70 eV. The melting points are uncorrected and were measured using an Electrothermal apparatus model 9100.

### Synthesis procedure

The synthesis was carried out in an undivided cylindrical glass cell (100 mL) containing NIC (1.0 mmol) and arylsulfinic acid (2.0 mmol) in a water (phosphate buffer, *c* = 0.2 M, pH = 6.0)/ethanol (50/50 v/v) mixture, equipped with a set of four graphite rods (each one 5.5 cm long and 10 mm in diameter) as anodes and a stainless steel rod as cathode, galvanostatically by applying a constant current density of 0.9 mA cm^−2^ (*I* = 20 mA) according to Table 2. At the end of the electrolysis, the solution was subjected to liquid–liquid extraction with 25 mL of ethyl acetate. After drying with magnesium sulfate and evaporation of the solvent, the resulting solid was subjected to thin layer chromatography (TLC) in a mixture of ethyl acetate and hexane (60/40). The solid obtained, after extensive washing with chloroform and complete evaporation, gave a yellow semi-crystalline precipitate that did not require further purification.

**Table 2:** Structure, isolated yield and electrolysis time of the synthesized compounds

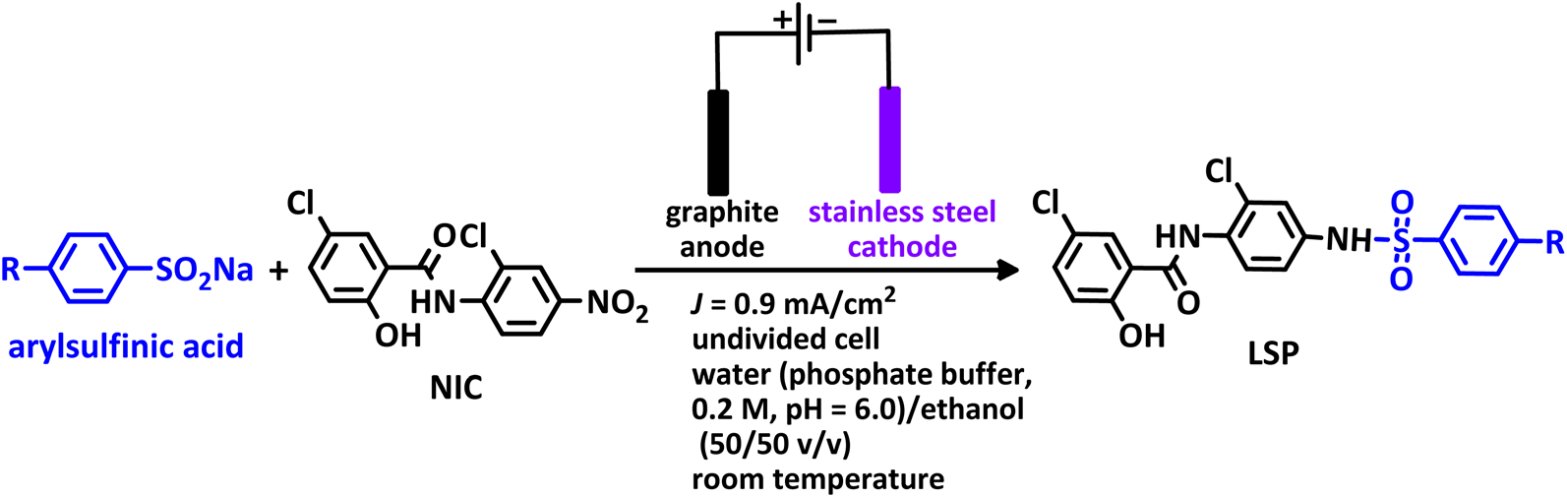				
Entry	Nucleophile	Electrolysis time (min)	Product	Isolated yield (%)
1	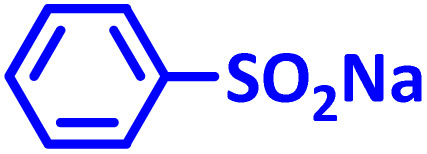	330	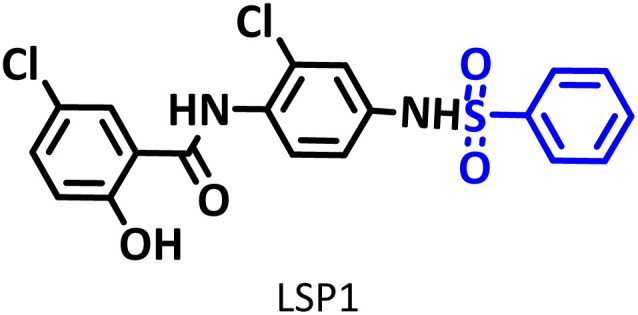	61
2	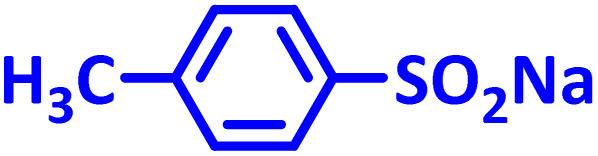	330	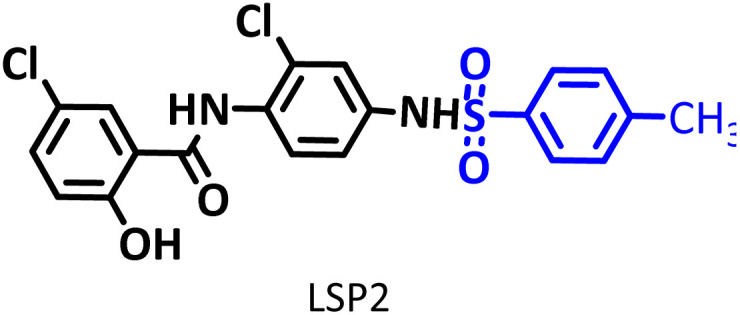	63
3	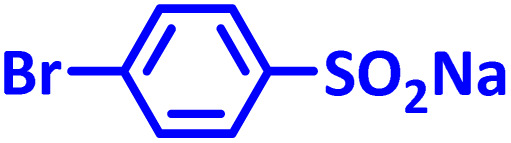	325	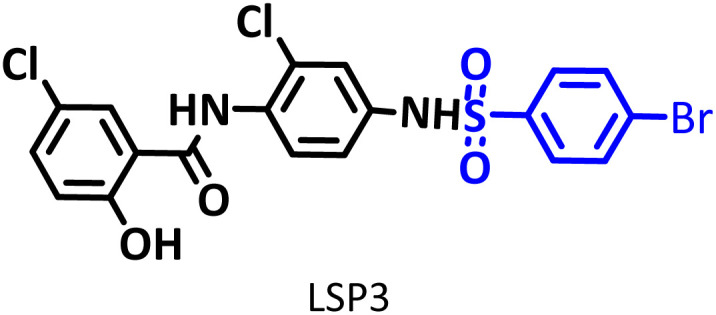	58

### Characteristics of the products

#### 5-Chloro-*N*-(2-chloro-4-(phenylsulfonamido)phenyl)-2-hydroxy benzamide (C_19_H_14_Cl_2_N_2_O_4_S) LSP1

Yellowish powder; mp 188–190 °C (dec.); ^1^H NMR, *δ* ppm (400 MHz, DMSO-*d*_6_): 7.13 (m, 3H, aromatic), 7.61 (m, 2H, aromatic), 7.78 (t, *J* = 8 Hz, 1H, aromatic), 7.98 (d, *J* = 3 Hz, 1H, aromatic), 8.31 (dd, *J* = 12 Hz and *J* = 4 Hz, 1H, aromatic), 8.39 (d, *J* = 12 Hz, 1H, aromatic), 8.45 (d, *J* = 2 Hz, 1H, aromatic), 8.83 (d, *J* = 9 Hz, 1H, aromatic), 10,92 (s, 1H, N–H),11.35 (s, 1H, N–H); ^13^C NMR, *δ* ppm (100 MHz, DMSO-*d*_6_): 119.7, 119.9, 121.3, 122.4, 122.9, 124.3, 124.4, 125.3, 126.0, 128.5, 130.5, 134.5, 141.6, 143.1, 155.6, 163.1; IR (KBr) (cm^−1^): 3581, 3498, 3096, 1681, 1655, 1631, 1605, 1581, 1563, 1419, 1347, 1327, 1289, 1194, 902, 745, 687; MS (*m*/*z*) (EI, 70 eV) (relative intensity): 436 (M^+^˙, 15), 282 (23), 156 (48), 141 (100), 99 (16), 77 (29).

#### 5-Chloro-*N*-(2-chloro-4-(4-methylphenylsulfonamido)phenyl)-2-hydroxybenzamide (C_20_H_16_Cl_2_N_2_O_4_S) LSP2

Cream powder; mp 194–196 °C (dec.); ^1^H NMR, *δ* ppm (400 MHz, DMSO-*d*_6_): 2.42 (s, 3H, CH_3_), 7.14 (m, 3H, aromatic), 7.54 (m, 2H, aromatic), 7.99 (d, *J* = 3.6 Hz, 1H, aromatic), 8.33 (dd, *J* = 12.0 Hz, *J* = 3.6 Hz, 1H, aromatic), 8.38 (d, *J* = 12 Hz, 1H, aromatic), 8.47 (d, *J* = 3.6 Hz, 1H, aromatic), 8.85 (d, *J* = 12.4 Hz, 1H, aromatic); 10.94 (s, 1H, N–H), 11.28 (s, 1H, N–H); ^13^C NMR, *δ* ppm (100 MHz, DMSO-*d*_6_): 21.6, 119.7, 120.0, 121.4, 122.5, 123.3, 124.2, 124.3, 125.2, 125.5, 129.9, 130.4, 134.4, 141.8, 143.1, 145.6, 155.9, 163.2; IR (KBr) (cm^−1^): 3403, 3107, 2927, 2851, 1709, 1607, 1541, 1346, 1326, 1197, 1104, 851, 817, 739, 678, 512; MS (*m*/*z*) (EI, 70 eV) (relative intensity): 450 (M^+^˙, 2), 326 (16), 296 (4), 246 (5), 192 (4), 155 (100), 127 (19), 99 (28), 63 (23).

#### *N*-(4-(4-Bromophenylsulfonamido)-2-chlorophenyl)-5-chloro-2-hydroxybenzamide (C_19_H_13_BrCl_2_N_2_O_4_S) LSP3

Light brown powder; mp 204–205 °C (dec.); ^1^H NMR, *δ* ppm (400 MHz, DMSO-*d*_6_): 7.07 (d, *J* = 11.6 Hz, 1H, aromatic), 7.32 (d, *J* = 11.2 Hz, 1H, aromatic), 7.47 (dd, *J* = 11.6, *J* = 3.6 Hz, 1H, aromatic), 7.52 (d, *J* = 11.6, 1H, aromatic), 7.70 (d, *J* = 11.2, 1H, aromatic), 7.84 (d, *J* = 11.6, 1H, aromatic), 7.92 (d, *J* = 4 Hz, 1H, aromatic), 8.27 (dd, *J* = 12.4, *J* = 3.6 Hz, 2H, aromatic), 8.40 (d, *J* = 3.2 Hz, 1H, aromatic), 8.84 (d, *J* = 12.4 Hz, 2H, aromatic), 12.0 (broad, 2H, N–H); ^13^C NMR, *δ* ppm (100 MHz, DMSO-*d*_6_): 119.7, 120.1, 121.0, 122.8, 123.0, 124.3, 125.2, 130.3, 133.2, 133.5, 134.3, 138.5, 142.1, 142.7, 157.4, 163.6; IR (KBr) (cm^−1^): 3452, 3272, 3086, 2926, 2859, 1654, 1567,1471, 1384, 1331, 1144, 1067, 1008, 917, 823, 725, 597, 505; MS (*m*/*z*) (EI, 70 eV) (relative intensity): 513 (M, 2), 326 (30), 266 (11), 223 (11), 180 (6), 155 (100), 127 (27), 99 (65), 63 (47).

## Data availability

The data from this paper, including [FT-IR, ^1^H NMR, ^13^C NMR, and MS spectra for compounds LSP1–LSP3] are available from the authors upon request.

## Conflicts of interest

The authors declare no conflict of interest.

## Supplementary Material

RA-015-D5RA02025E-s001
