# Supplementary material for: Successive paired electrochemical late-stage modification of niclosamide a common anthelmintic drug. A green protocol for the synthesis of new drug-like molecules
Source: RSC Adv. 2025 May 29;15(22):17803–10. doi: 10.1039/d5ra02025e (PMC12120933; doi:10.1039/d5ra02025e)

Supplementary Information (SI) for RSC Advances.
This journal is © The Royal Society of Chemistry 2025

Supplementary Information

Successive Paired Electrochemical Late-Stage Modification of Niclosamide a Common Anthelmintic Drug. A Green Protocol for the Synthesis of New Drug-Like Molecules

Haniya Rezaei,[a] Davood Nematollahi,[a,b*] Niloofar Mohamadighader,[a] Farideh Lotfipour[a]

[a]Faculty of Chemistry and Petroleum Sciences, Bu-Ali Sina University, Hamedan, Iran. Zip Code 65178-38683.

[b]Planet Chemistry Research Center, Bu-Ali Sina University, Hamedan, Iran.

E-mail addresses: *nemat@basu.ac.ir, dnematollahi@yahoo.com* (D. Nematollahi).

Fax: +0098 813 8257407, Tel: +0098 813 8282807.

Contents

FT-IR spectrum of **LSP1**......1

^{1}H NMR spectrum of **LSP1** 2

Expanded 1H NMR spectrum of **LSP1**...... 3

^{13}C NMR spectrum of **LSP1** 4

Expanded ^{13}C NMR spectrum of **LSP1**...... 5

MS spectrum of **LSP1** 6

FT-IR spectrum of **LSP2** 7

^{1}H NMR spectrum of **LSP2** 8

Expanded ^{1}H NMR spectrum of **LSP2**...... 9

^{13}C NMR spectrum of **LSP2** 10

Expanded ^{13}C NMR spectrum of **LSP2**...... 11

MS spectrum of **LSP2** 12

FT-IR spectrum of **LSP3** 13

^{1}H NMR spectrum of **LSP3** 14

Expanded ^{1}H NMR spectrum of **LSP3**...... 15

^{13}C NMR spectrum of **LSP3** 16

Expanded ^{13}C NMR spectrum of **LSP3**...... 17

MS spectrum of **LSP3** 18

FT-IR spectrum of LSP1

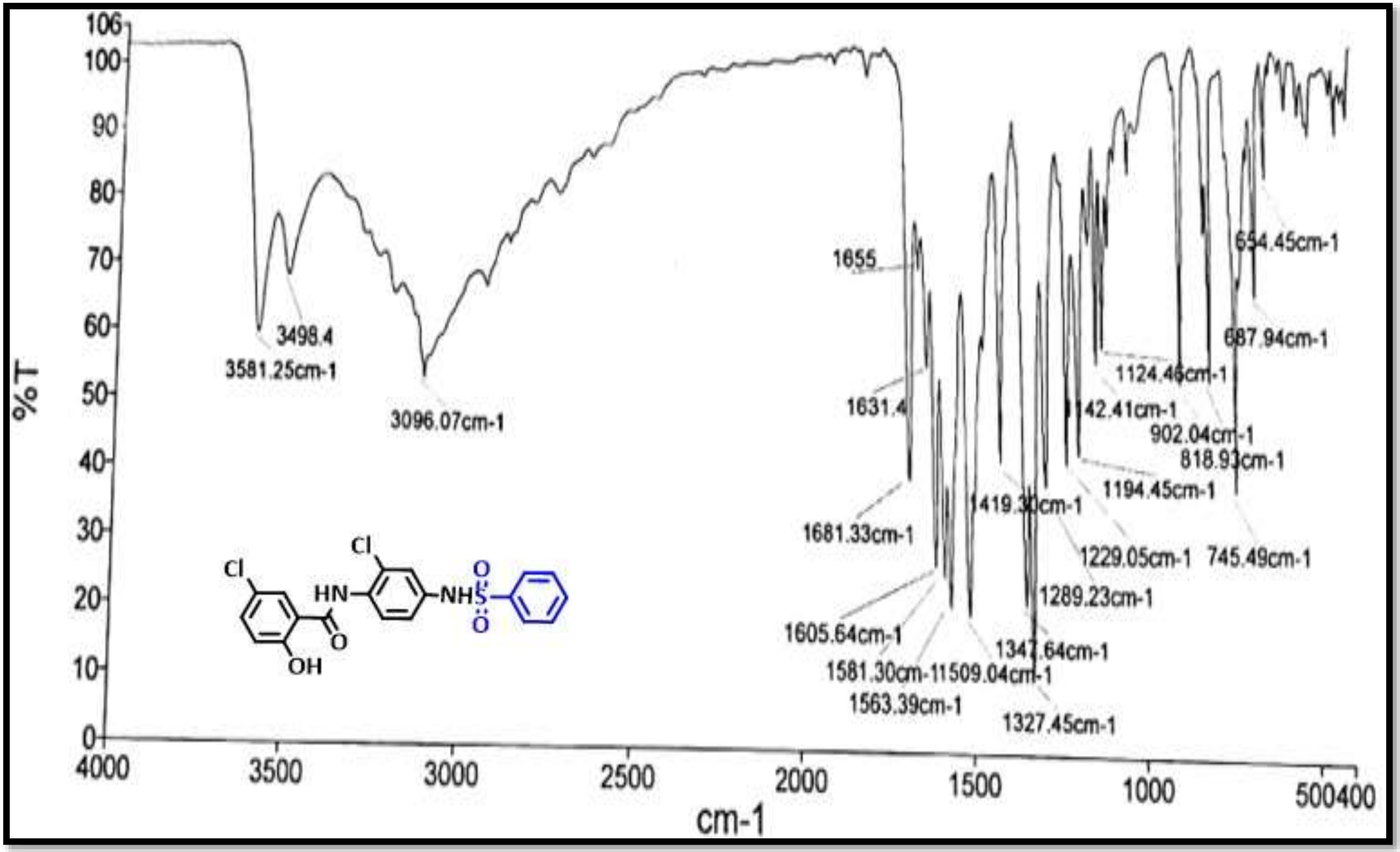

^{1}H NMR spectrum of LSP1

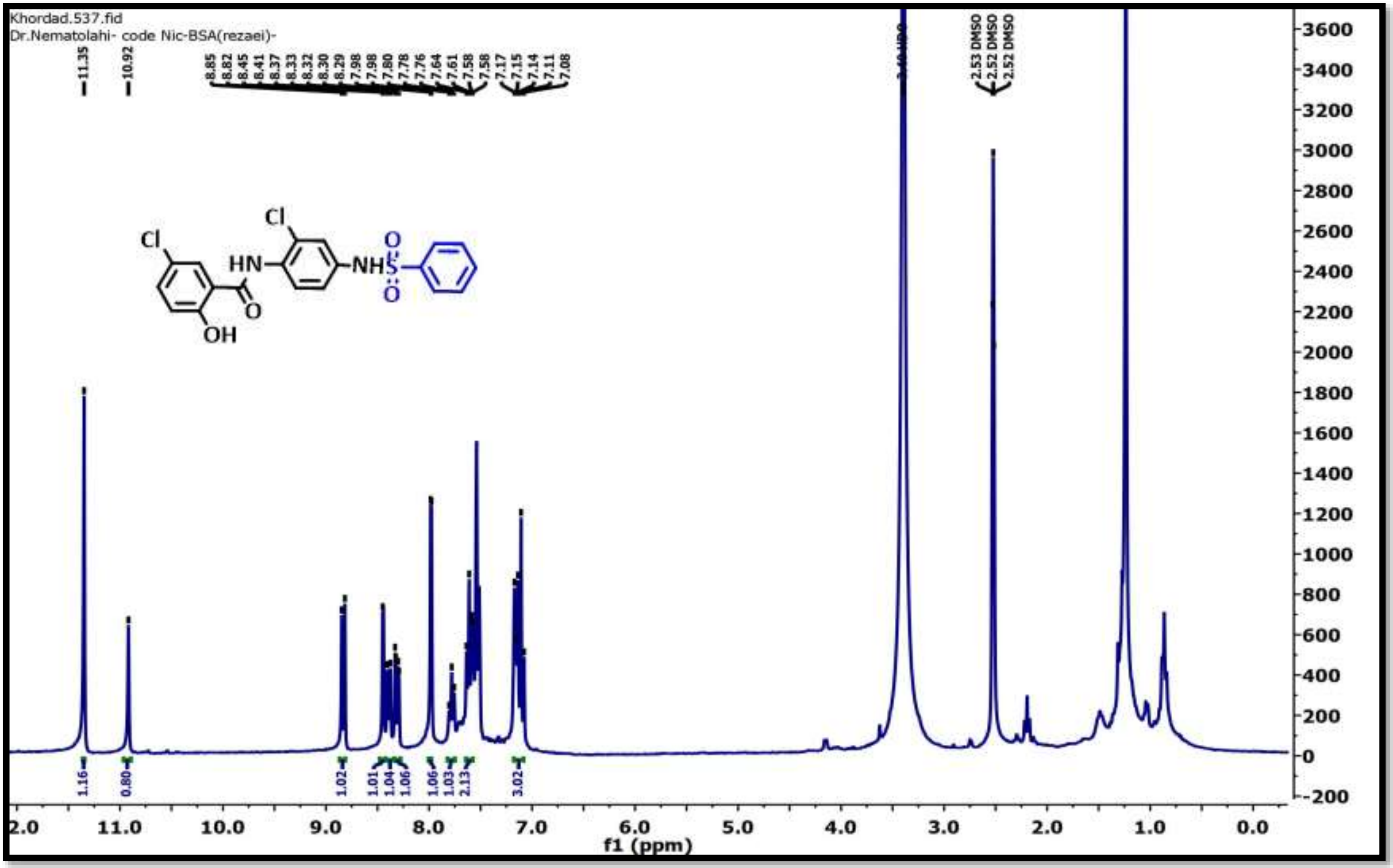

Expanded ^{1}H NMR spectrum of LSP1

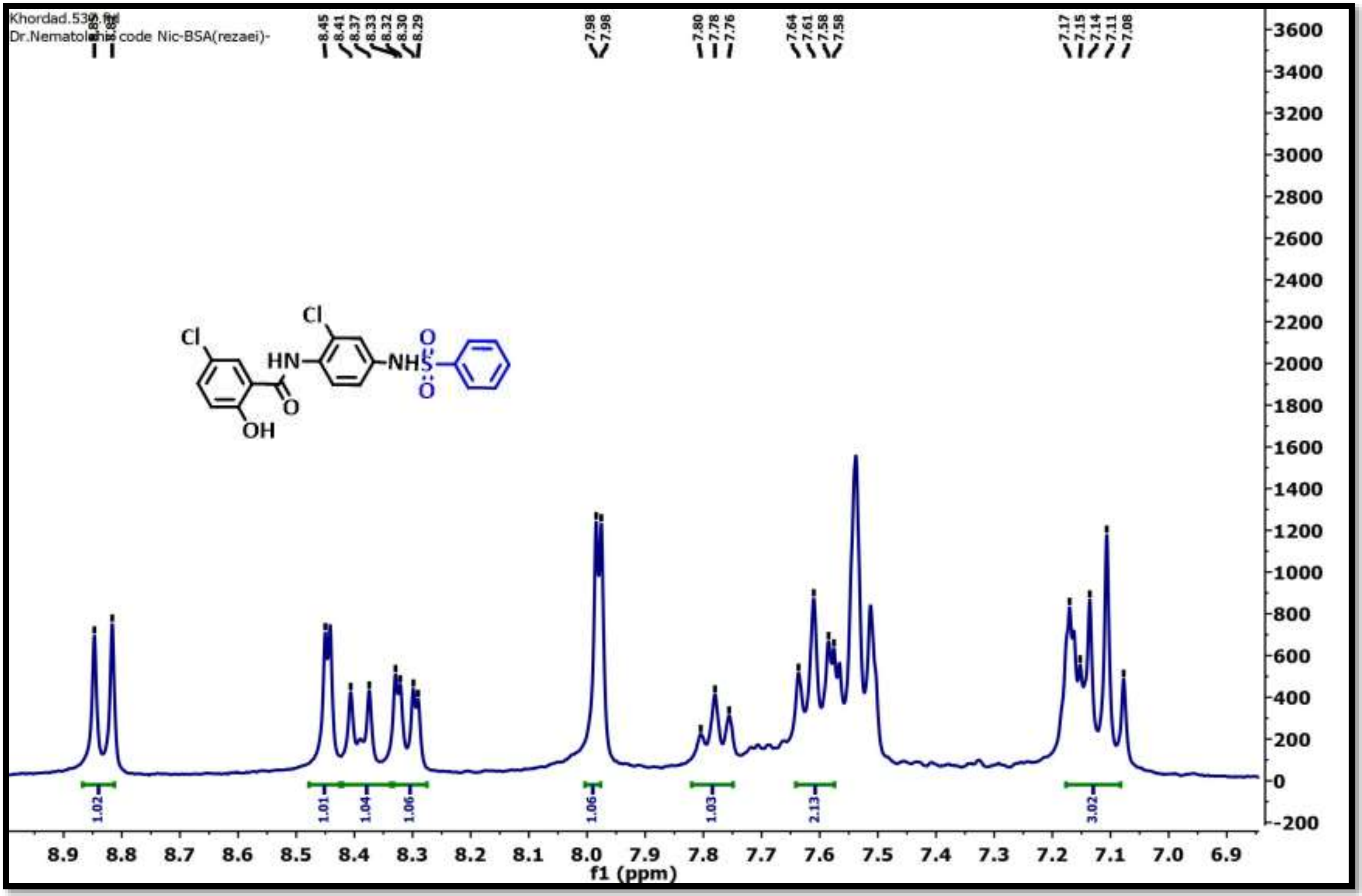

^{1}H NMR, δ ppm (400 MHz, DMSO-d_6)**:** 7.13 (m, 3H, aromatic), 7.61 (m, 2H, aromatic), 7.78 (t, *J* = 8 Hz 1H, aromatic), 7.98 (d, *J* = 3 Hz, 1H, aromatic), 8.31 (dd, *J* = 12 Hz and *J* = 4 Hz, 1H, aromatic), 8.39 (d, *J* = 12 Hz, 1H, aromatic), 8.45 (d, *J* = 2 Hz, 1H, aromatic), 8.83 (d, *J* = 9 Hz, 1H, aromatic), 10,92 (s, 1H, N-H),11.35 (s, 1H, N-H).

^{13}C NMR spectrum of LSP1

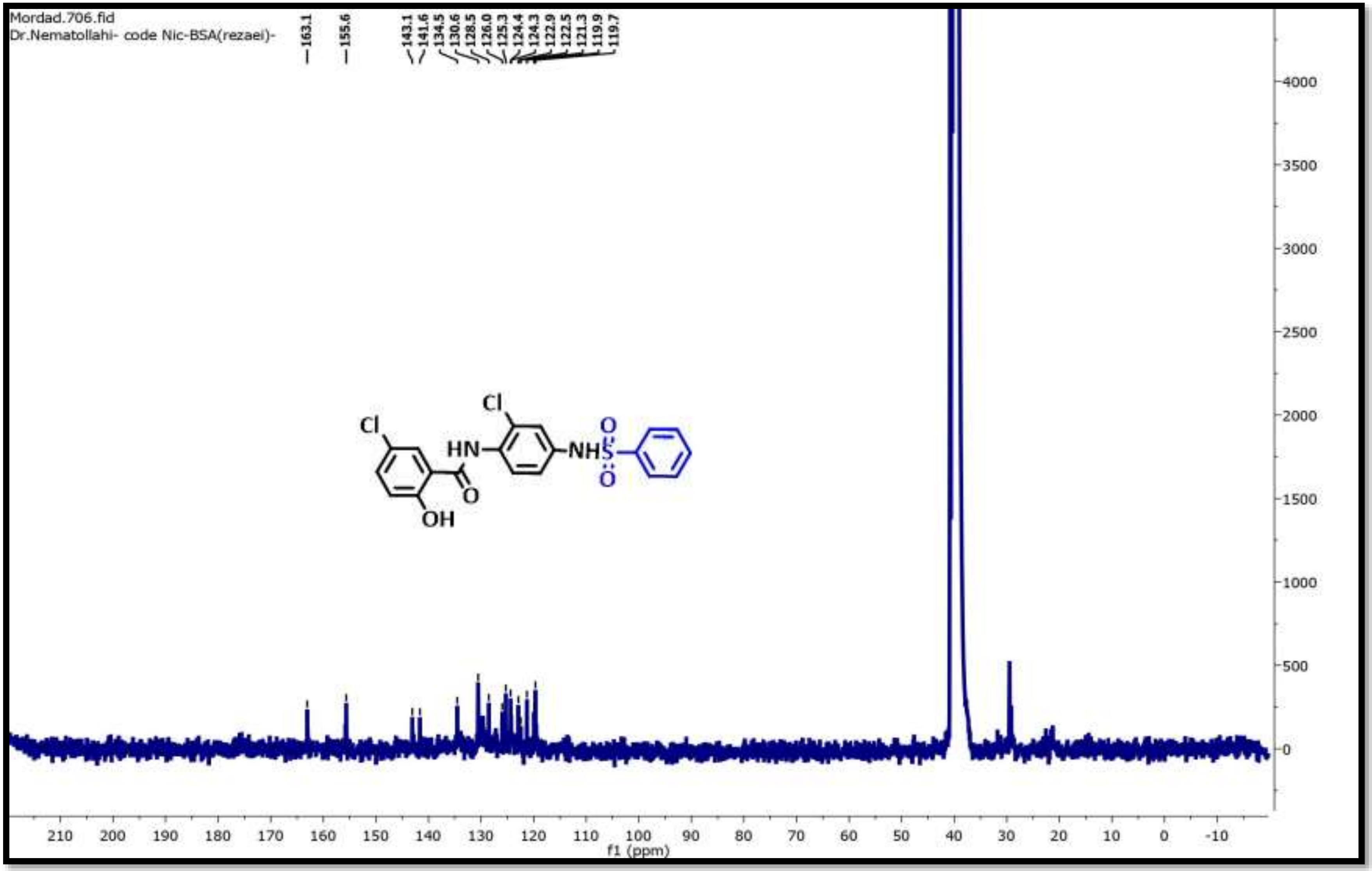

Expanded ^{13}C NMR spectrum of LSP1

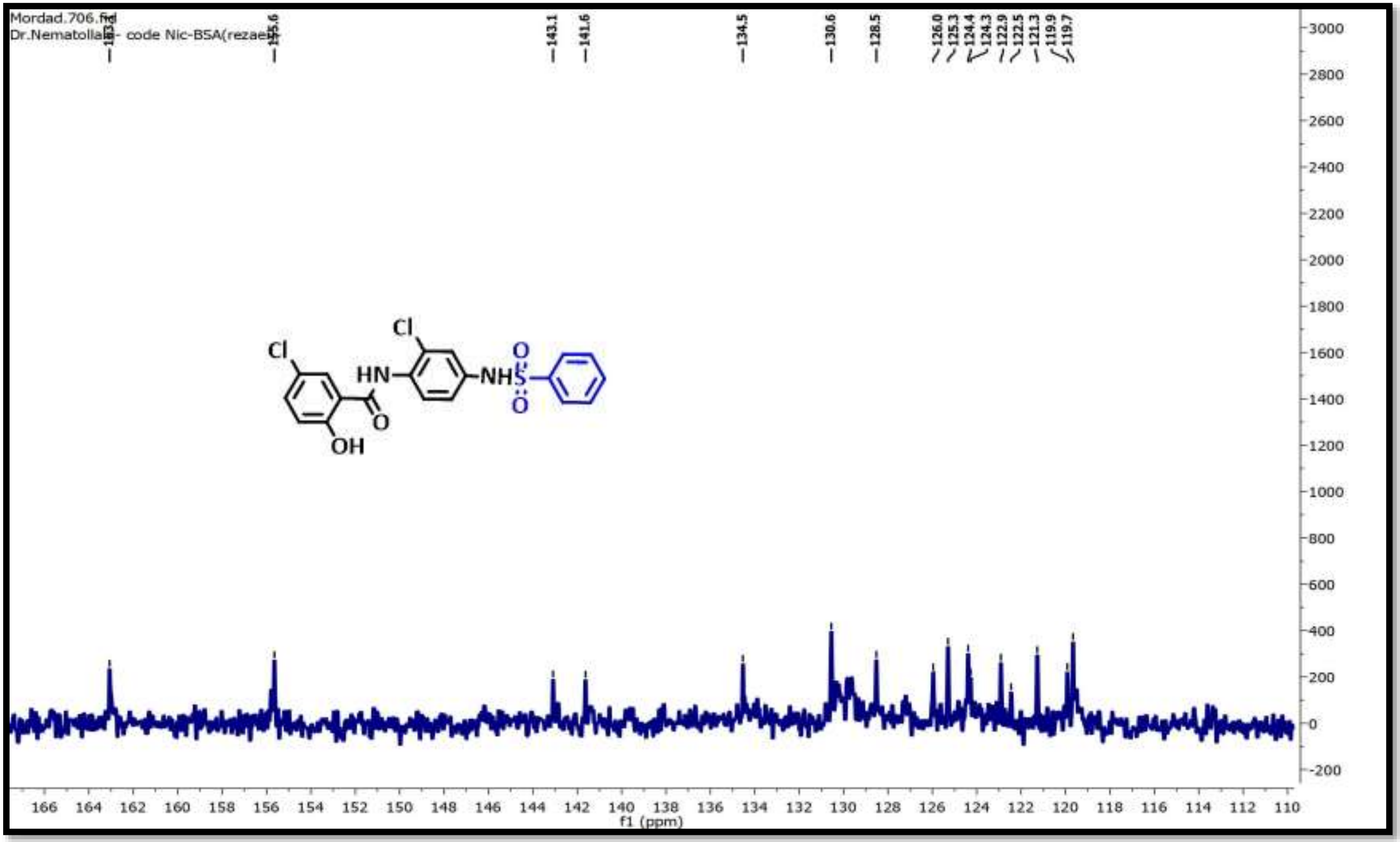

MS spectrum of LSP1

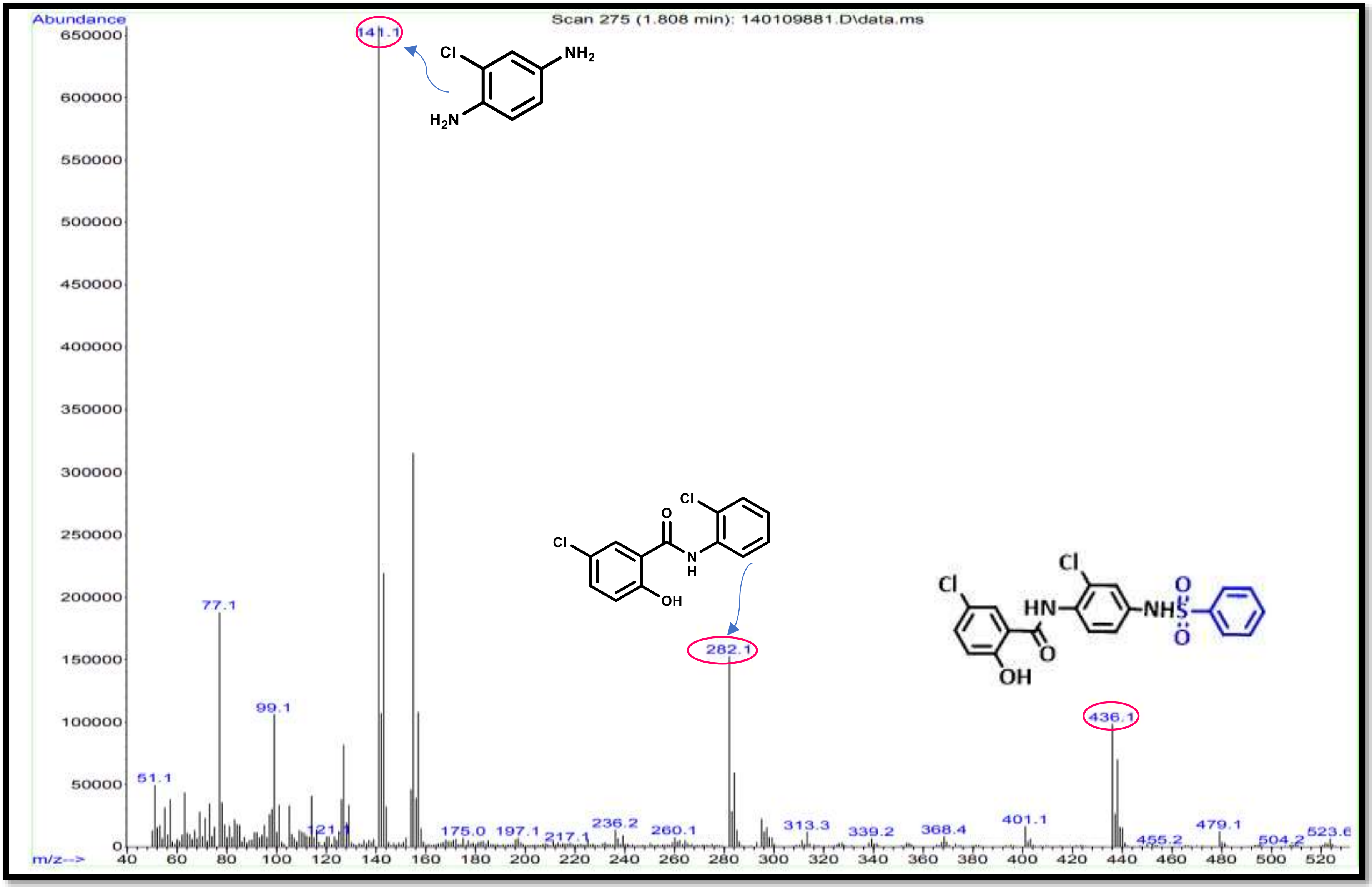

FT-IR spectrum of LSP2

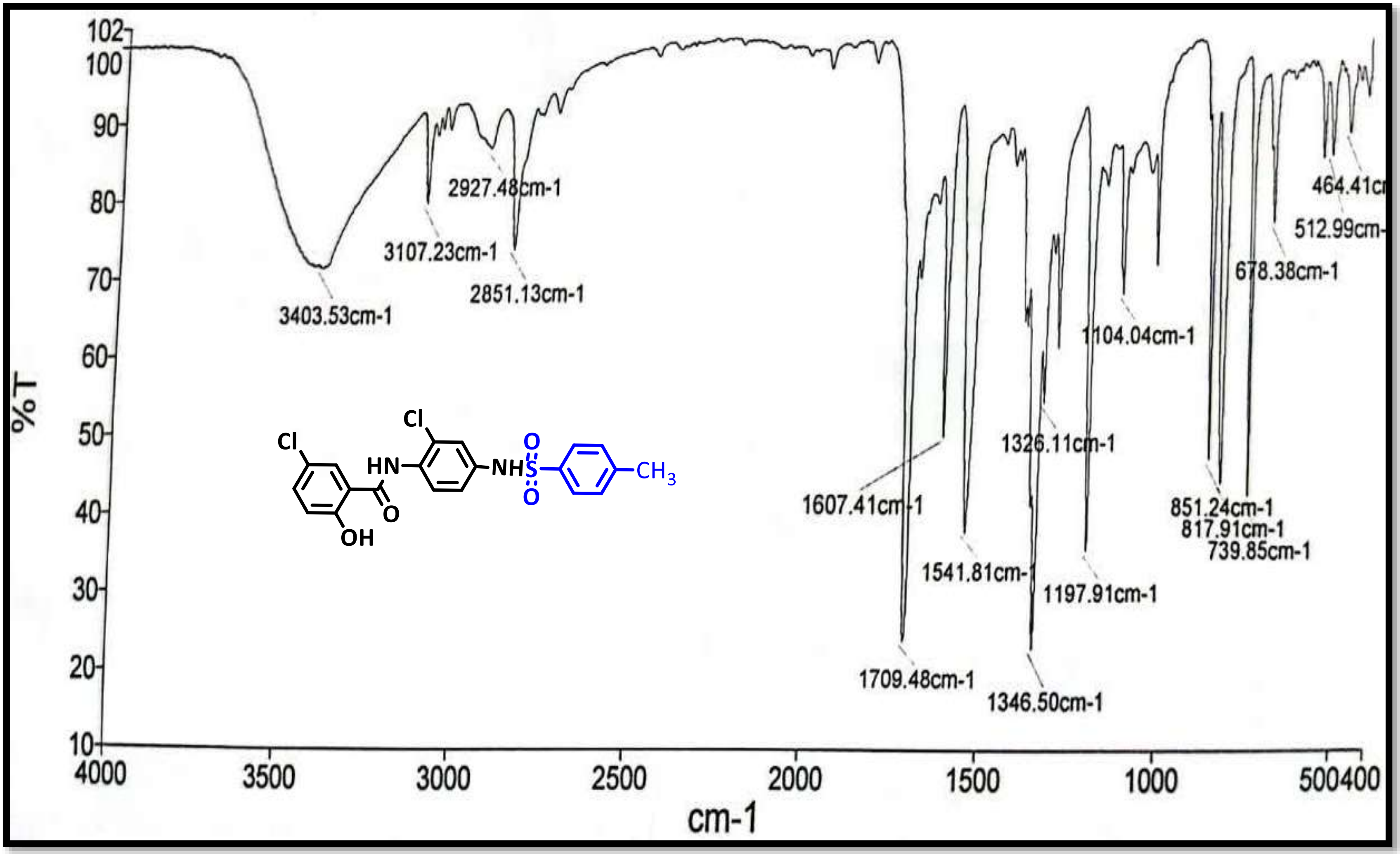

^{1}H NMR spectrum of LSP2

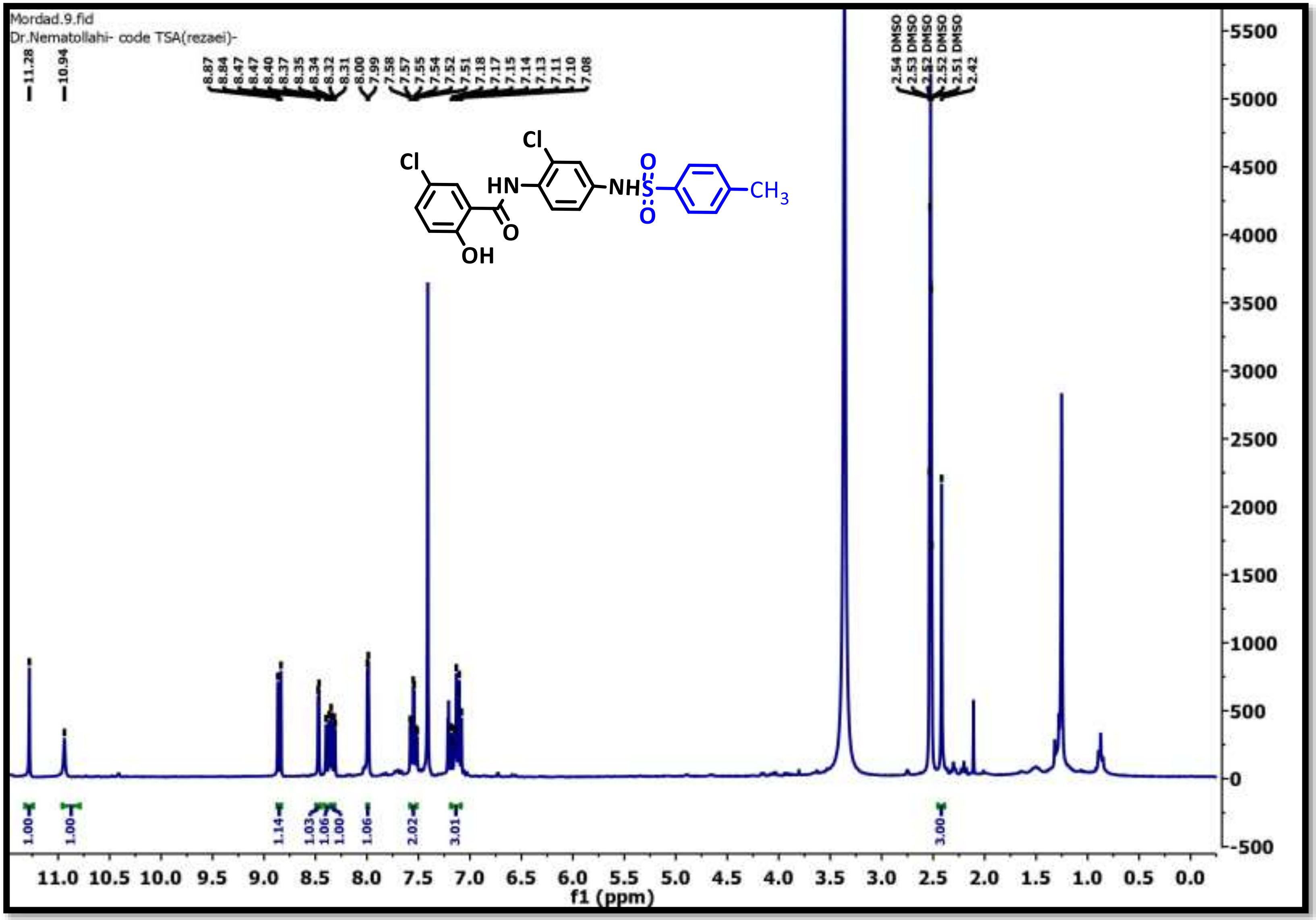

Expanded ^{1}H NMR spectrum of LSP2

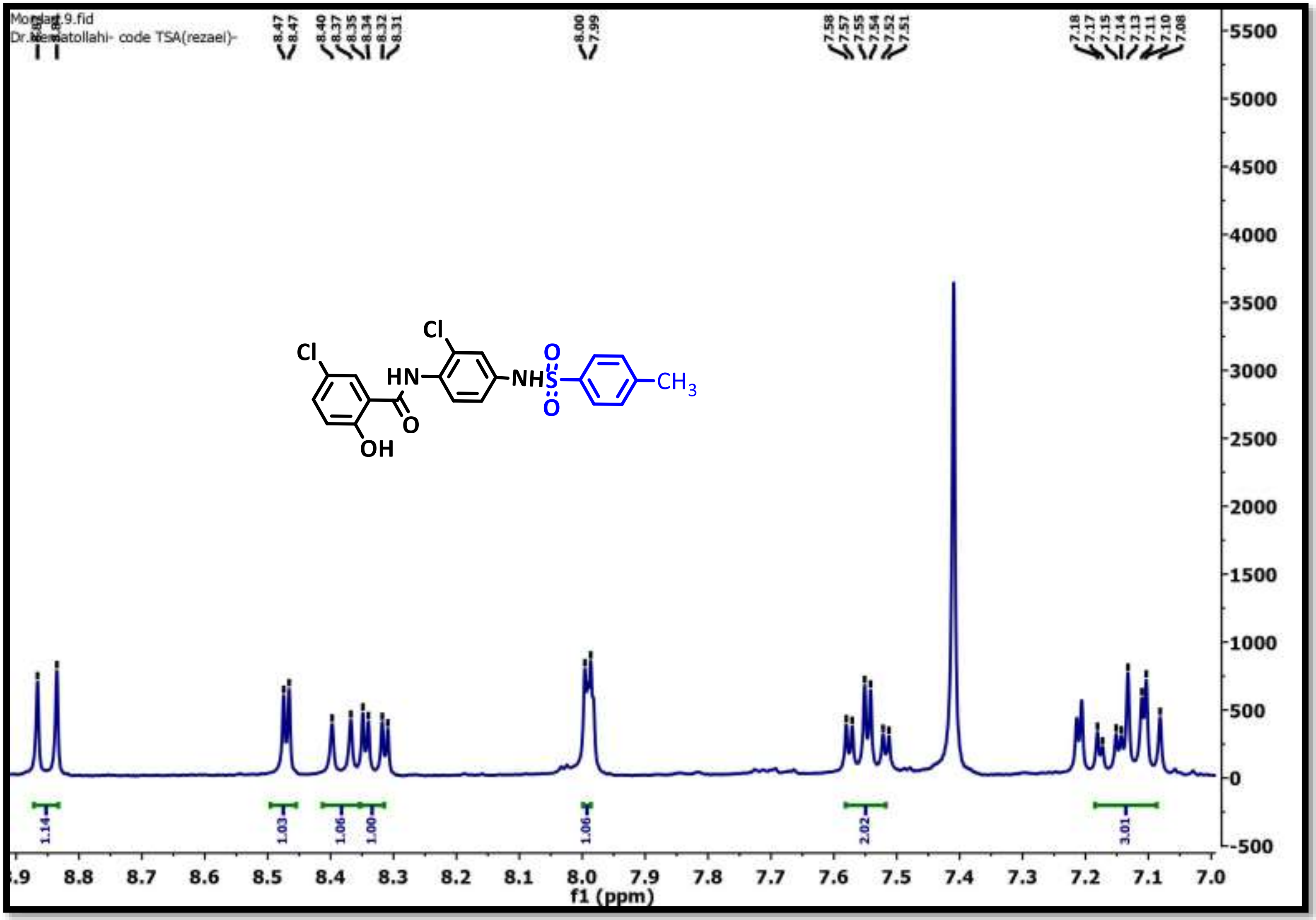

^1H NMR, δ ppm (400 MHz, DMSO-d_6)***:*** 2.42 (s, 3H, CH_3), 7.14 (m, 3H, aromatic), 7.54 (m, 2H, aromatic), 7.99 (d, J = 3.6, 1H, aromatic), 8.33 (dd, J = 12.0 Hz, J = 3.6 Hz, 1H, aromatic), 8.38 (d, J = 12 Hz, 1H, aromatic), 8.47 (d, J = 3.6 Hz, 1H, aromatic), 8.85 (d, J = 12.4 Hz, 1H, aromatic); 10.94 (s, 1H, N-H), 11.28 (s, 1H, N-H).

^{13}C NMR spectrum of LSP2

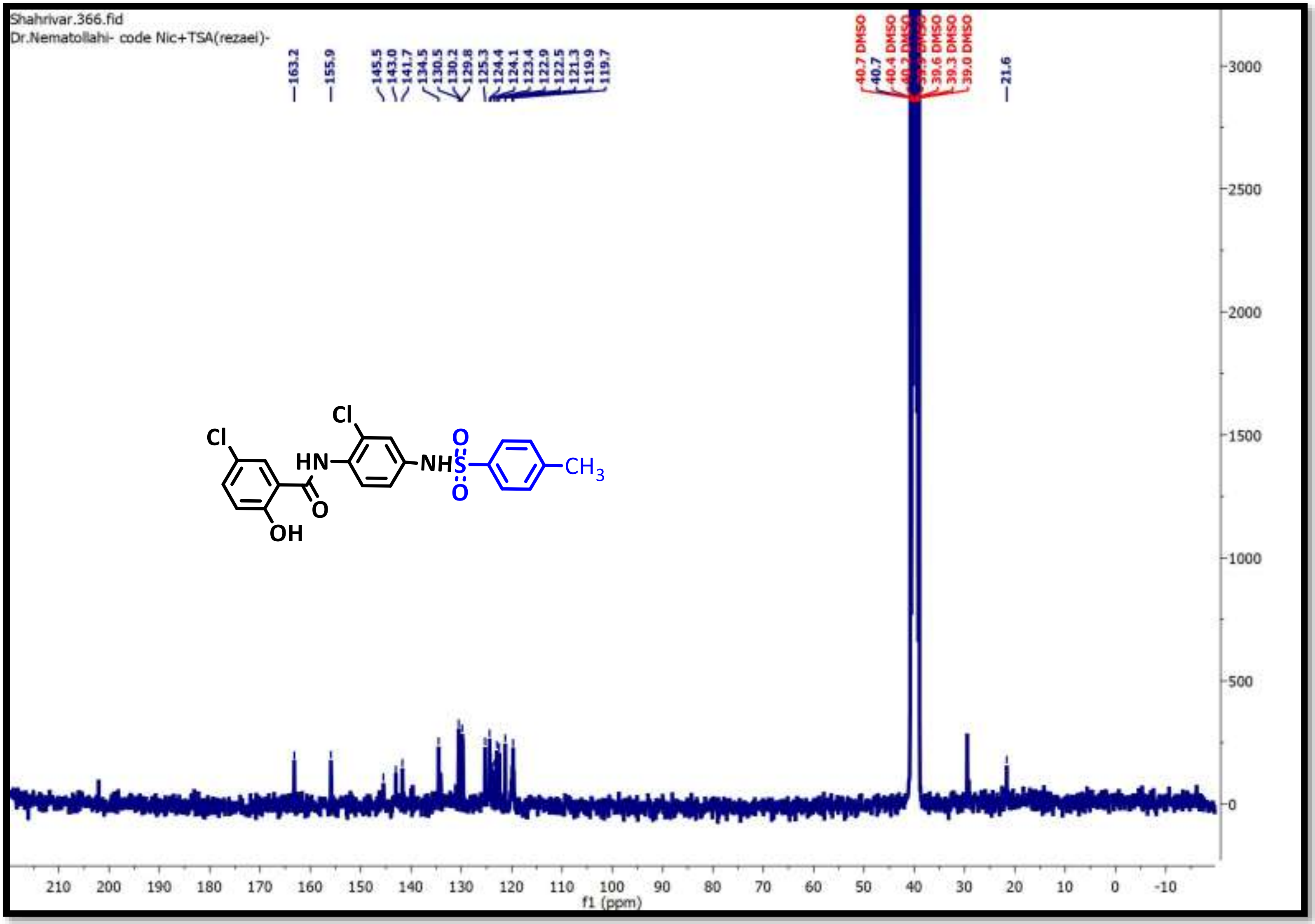

Expanded ^{13}C NMR spectrum of LSP2

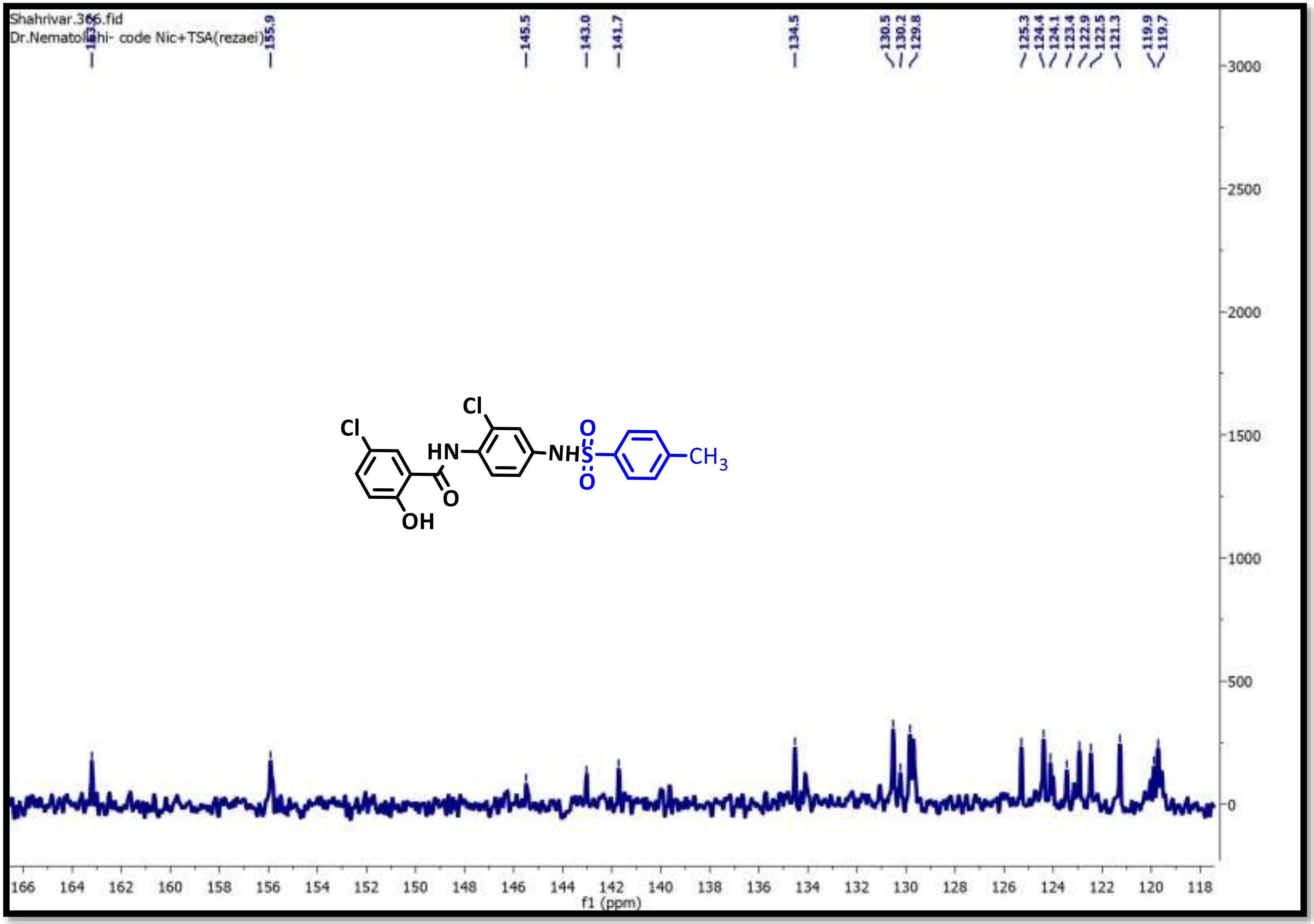

MS spectrum of LSP2

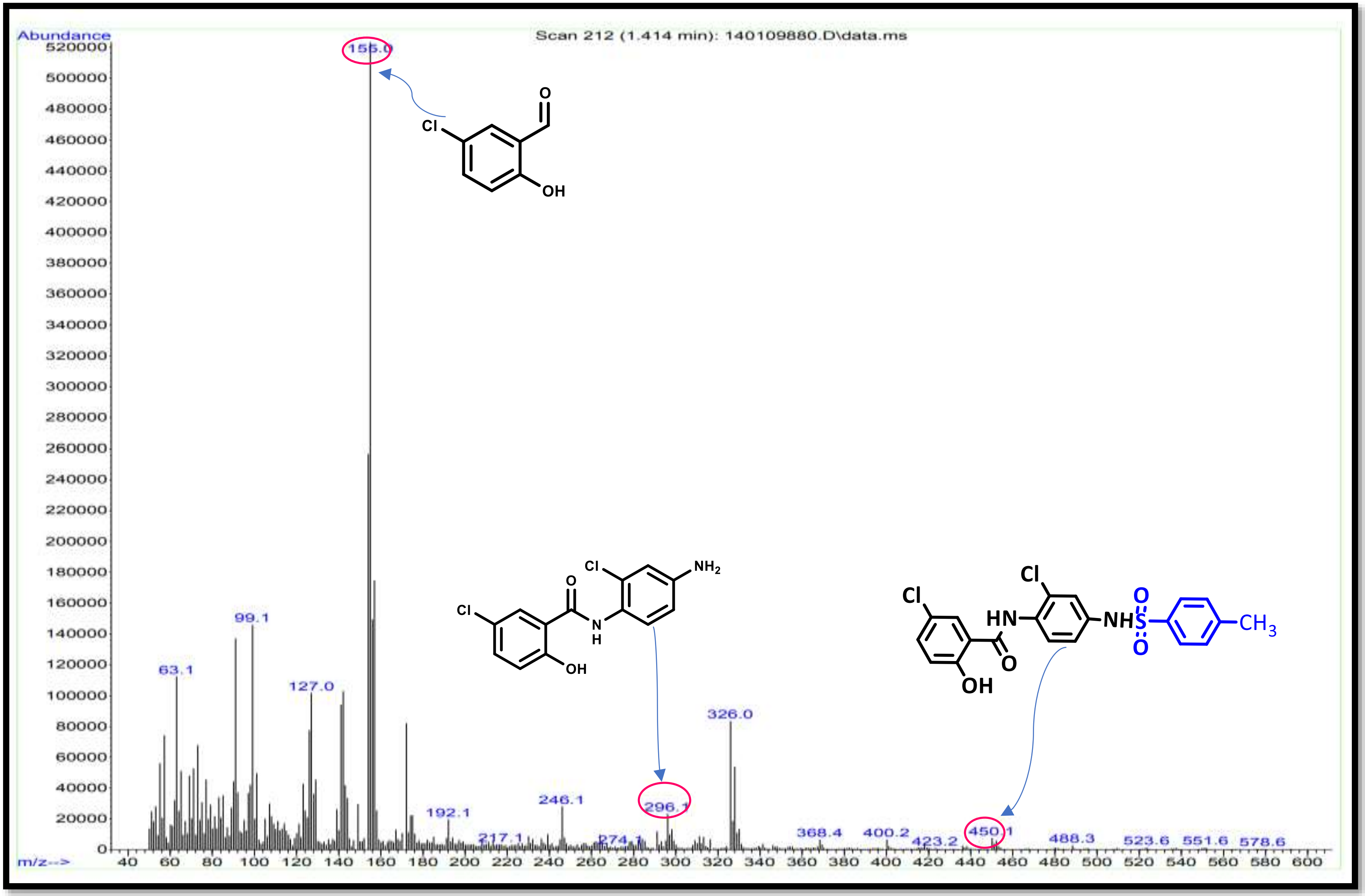

FT-IR spectrum of LSP3

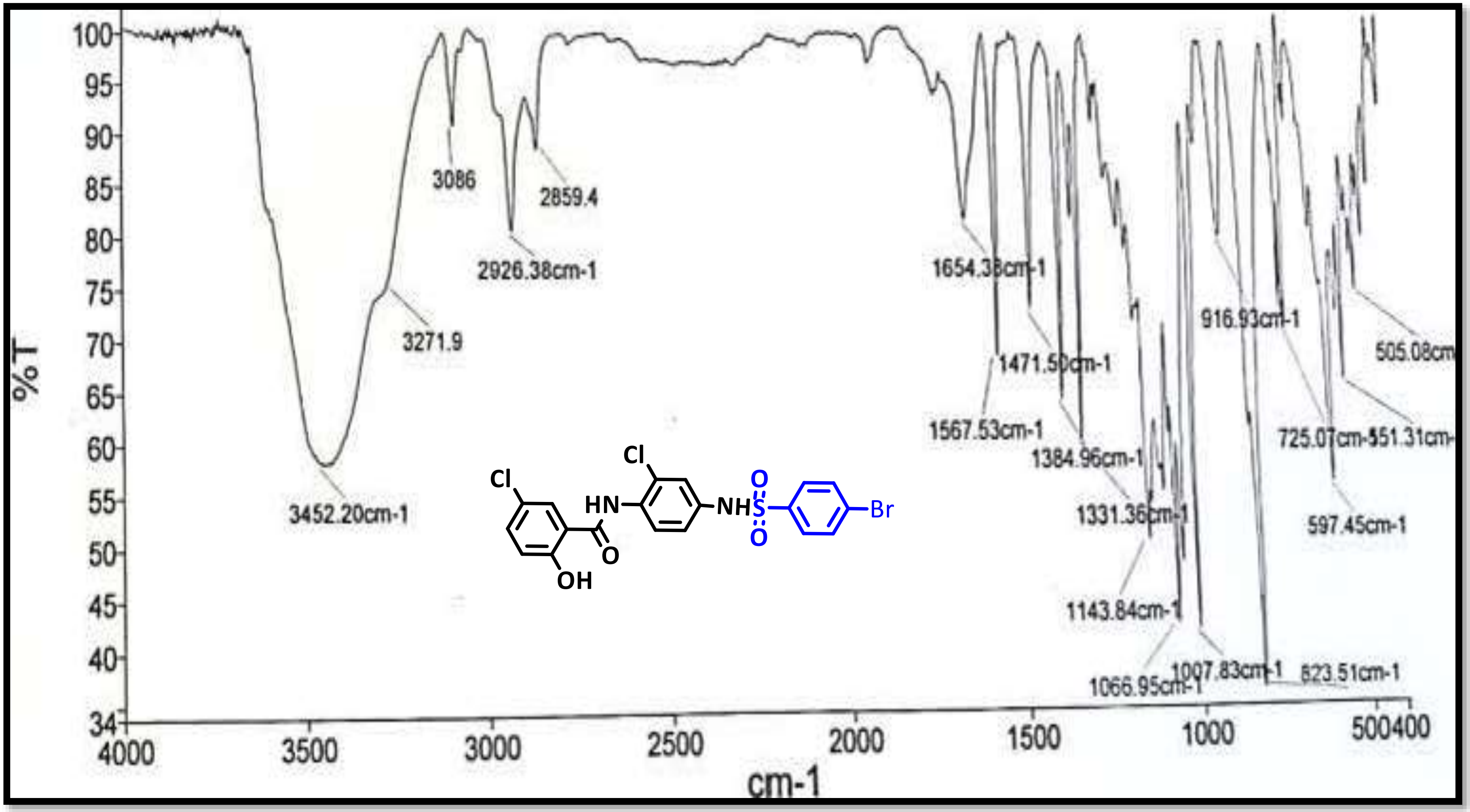

^{1}H NMR spectrum of LSP3

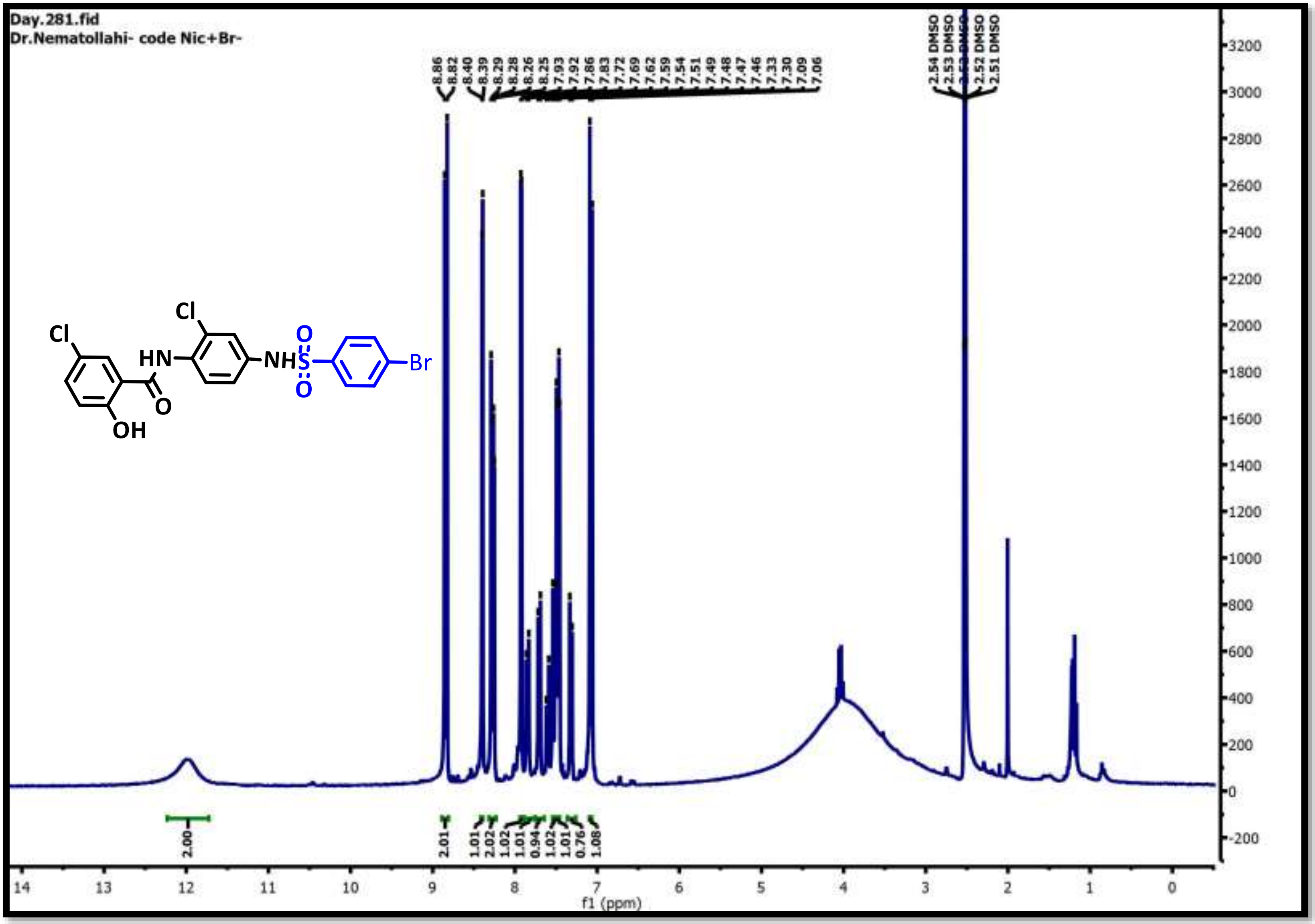

Expanded ^{1}H NMR spectrum of LSP3

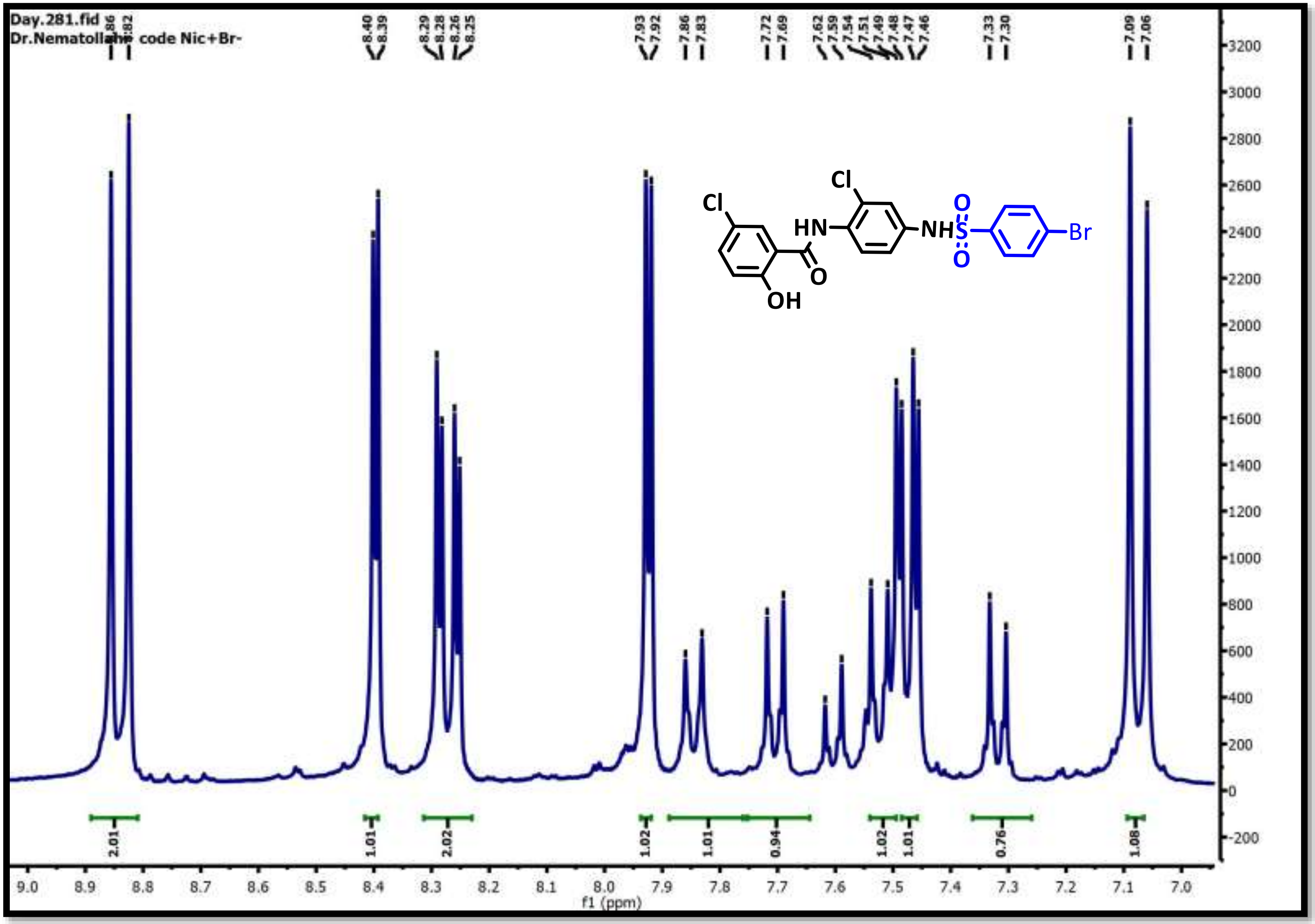

^1H NMR, δ ppm (400 MHz, DMSO-d_6)***:*** 7.07 (d, *J* =11.6 Hz, 1H, aromatic), 7.32 (d, *J* =11.2 Hz, 1H, aromatic), 7.47 (dd, *J* = 11.6, *J* = 3.6 Hz, 1H, aromatic), 7.52 (d, *J* = 11.6, 1H, aromatic), 7.70 (d, *J* = 11.2, 1H, aromatic), 7.84 (d, *J* = 11.6, 1H, aromatic), 7.92 (d, *J* = 4 Hz, 1H, aromatic), 8.27 (dd, *J* = 12.4, *J* = 3.6 Hz, 2H, aromatic), 8.40 (d, *J* = 3.2 Hz, 1H, aromatic), 8.84 (d, *J* = 12.4 Hz, 2H, aromatic), 12.0 (broad, 2H, N-H).

^{13}C NMR spectrum of LSP3

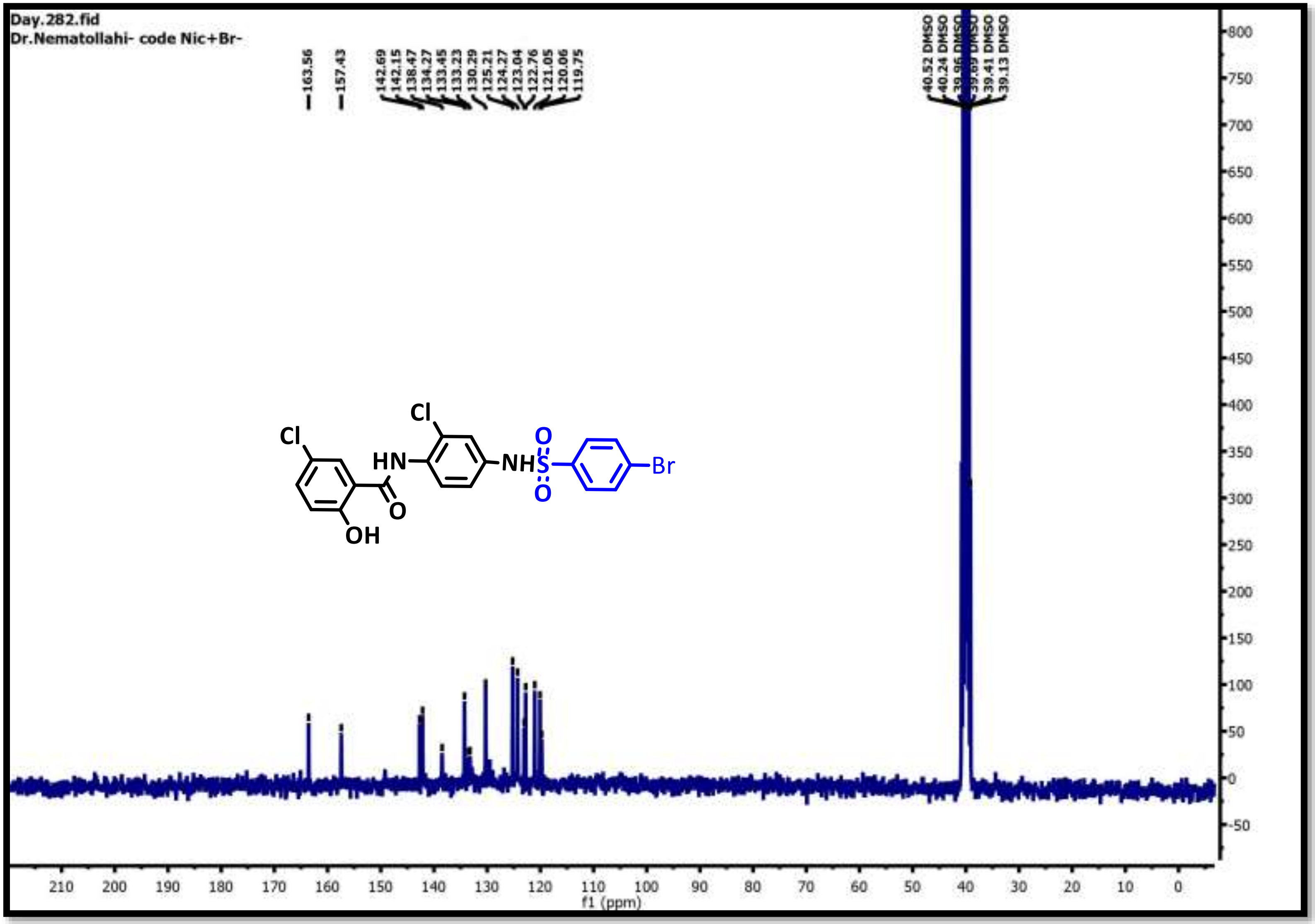

Expanded ^{13}C NMR spectrum of LSP3

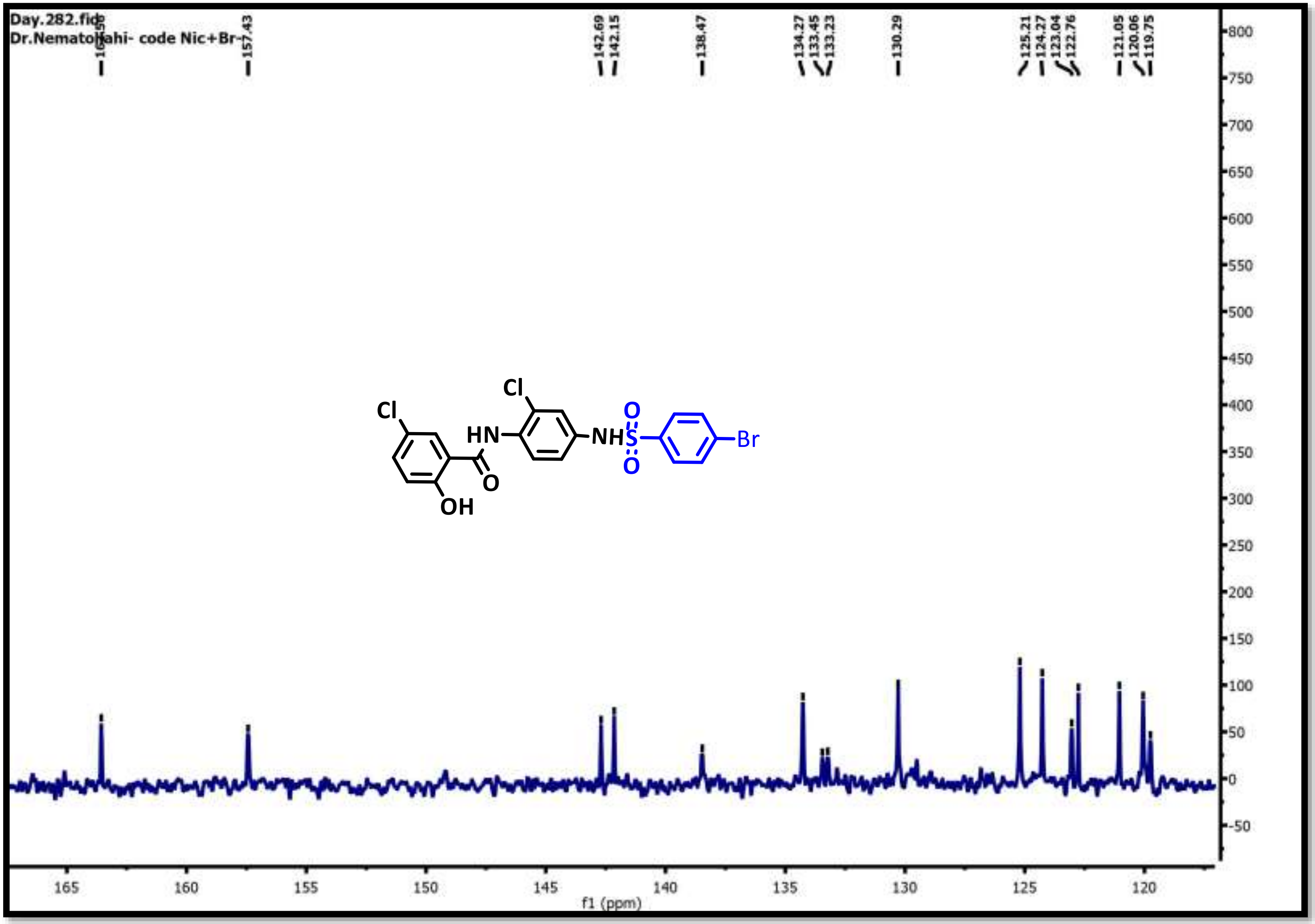

MS spectrum of LSP3

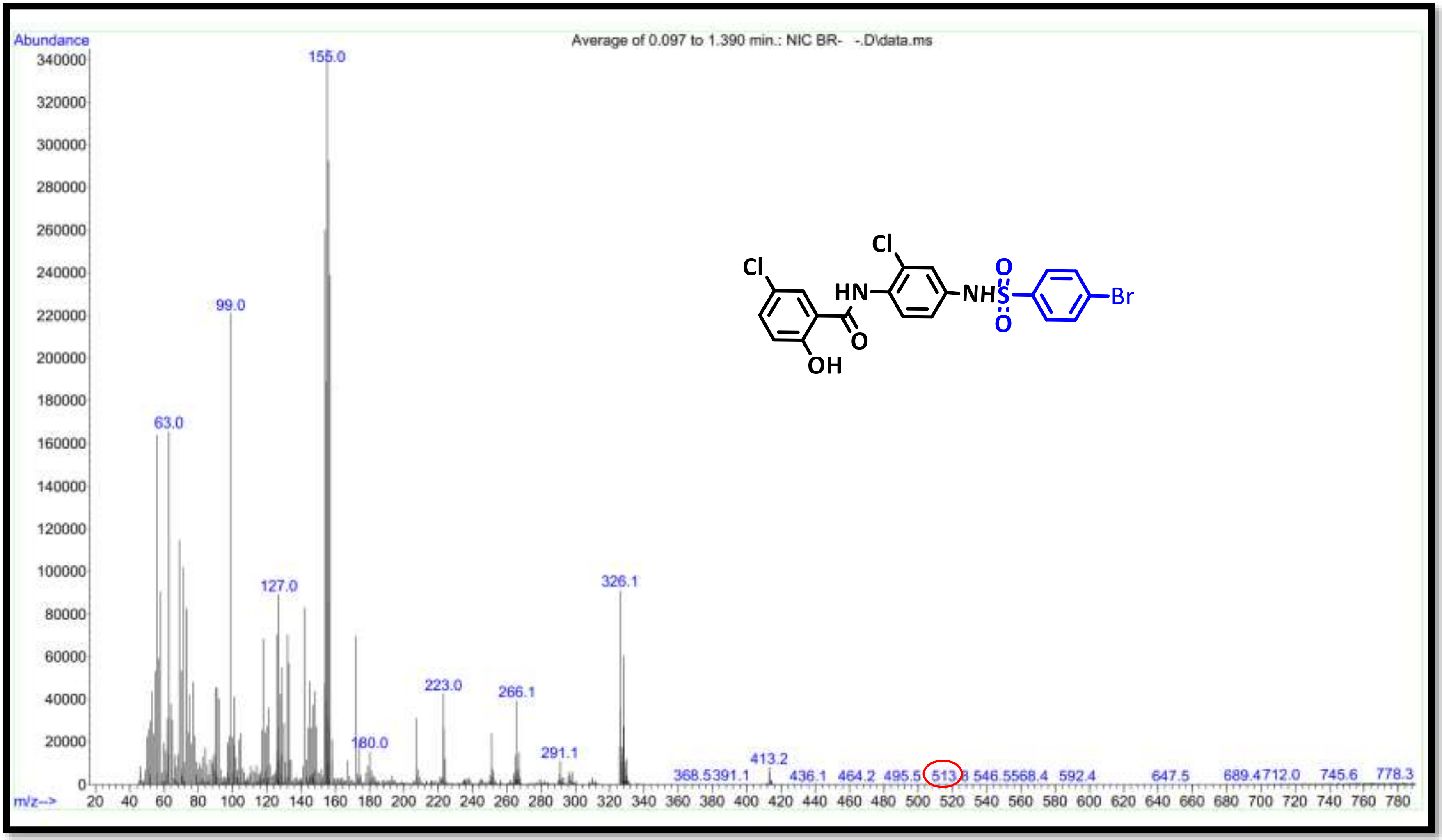

Supplement: RA-015-D5RA02025E-s001 [file RA-015-D5RA02025E-s001.pdf]
